# Mechanistic insight for improving butenyl-spinosyn production through combined ARTP/UV mutagenesis and ribosome engineering in *Saccharopolyspora pogona*

**DOI:** 10.3389/fbioe.2023.1329859

**Published:** 2024-01-15

**Authors:** Xueli Zhao, Muhammad Hammad Hussain, Ali Mohsin, Zebo Liu, Zhixian Xu, Zhanxia Li, Weiqun Guo, Meijin Guo

**Affiliations:** ^1^ State Key Laboratory of Bioreactor Engineering, East China University of Science and Technology, Shanghai, China; ^2^ Department of Respiratory Medicine, Shanghai Sixth People’s Hospital Affiliated to Shanghai Jiao Tong University School of Medicine, Shanghai, China; ^3^ Academy of National Food and Strategic Reserves Administration, Beijing, China

**Keywords:** *Saccharopolyspora pogona*, butenyl-spinosyn, ARTP/UV mutagenesis, ribosome engineering, fermentation process, multi-omics analysis

## Abstract

Butenyl-spinosyn is a highly effective, wide-spectrum and environmentally-friendly biological insecticide produced by *Saccharopolyspora pogona*. However, its scale-up is impeded due to its lower titer in wild-type strains. In this work, ARTP/UV mutagenesis and ribosome engineering were employed to enhance the butenyl-spinosyn production, and a stable mutant *Saccharopolyspora pogona* aG6 with high butenyl-spinosyn yield was successfully obtained. For the first time, the fermentation results in the 5 L bioreactor demonstrated that the butenyl-spinosyn produced by mutant *Saccharopolyspora pogona* aG6 reached the maximum value of 130 mg/L, almost 4-fold increase over the wild-type strain WT. Furthermore, comparative genomic, transcriptome and target metabolomic analysis revealed that the accumulation of butenyl-spinosyn was promoted by alterations in ribosomal proteins, branched-chain amino acid degradation and oxidative phosphorylation. Conclusively, the proposed model of ribosome engineering combined with ARTP/UV showed the improved biosynthesis regulation of butenyl-spinosyn in *S. pogona*.

## 1 Introduction

Butenyl-spinosyn, produced in the actinomycete *Saccharopolyspora pogona* (*S. pogona*), has a similar macrolide structure to spinosyn and thus exhibits insecticidal, acaricidal, and parasiticide activities (Li et al., 2022; Rang et al., 2022). Compared to spinosyn, butenyl-spinosyn possesses a wider insecticidal spectrum and it is more environmentally sustainable than spinosyn (Rang et al., 2020; He et al., 2021a). For instance, butenyl-spinosyn exhibits outstanding efficiency in controlling apple moths and tobacco worms. Nevertheless, the yield of butenyl-spinosyn in the wild-type *S. pogona* is significantly low, limiting its popularity and industrialization (Tang et al., 2021). In recent decades, considerable efforts have been devoted through genetic manipulation to enhance the production of butenyl-spinosyn in *S. pogona*. Deleting the flaviolin-like gene cluster in *S. pogona* resulted in a 4.06-fold increase in the production of butenyl-spinosyn, reaching 107.3 mg/L in shake flask experiments (He et al., 2021b). Overexpressed polynucleotide phosphorylase gene using a strong ermE* promoter, which resulted in a 1.92-fold increase in butenyl-spinosyn yield (Li et al., 2018). However, the challenges associated with the molecular modification of *S. pogona* and the complexity of its regulation network are significant challenges that hinder the production of butenyl-spinosyn at the yields required for large-scale agricultural applications (Rang et al., 2021). Hence, there is a dire need to improve the current low yield of butenyl-spinosyn.

Random mutagenesis techniques, such as UV irradiation and atmospheric and room temperature plasma (ARTP), are significant in strain improvement (Song et al., 2017). UV irradiation, which induces a broad spectrum of point mutations (Khaliq et al., 2009), is a traditional and successful mutagenesis method. Compared with the chemical method, UV mutagenesis offers the advantage of being more flexible and potentially allowing for better control to avoid secondary contamination (Liu et al., 2015). ARTP has been shown to cause a variety of changes in plasmid DNA and oligonucleotides by generation of plasma jets at room temperature and pressure, including missense mutations, deletions, and nucleic frameshift mutations (Wang et al., 2014). ARTP, known for its higher mutation rate, environmental friendliness, and ease of operation, stands as a potent tool for random mutagenesis. However, each mutagenesis method has its own specific mutagenesis mechanisms and limitations. Therefore, the combination of different mutagenesis methods could increase the diversity of mutations and help to improve the mutagenesis efficiency.

In addition to the random mutagenesis that increases antibiotic production, the ribosome engineering technique was also a powerful method to enhance the yield of antibiotics (Tanaka et al., 2017). Antibiotic-induced ribosome engineering could result in genetic code alterations of ribosomes and RNA polymerases. For instance, streptomycin targets the 30S ribosomal protein S12, leading to resistance mutations (Lopatniuk et al., 2019). Paromomycin induces resistance mutations at the 23S rRNA subunit (Zhang et al., 2023). This process modulated gene expression, subsequently influencing networks and the biosynthesis of secondary metabolites (Zhang et al., 2019). Interestingly, in previous studies, the ribosome engineering technique was also integrated with random mutagenesis to achieve better results in terms of effectiveness and desired outcomes. Combination of UV mutagenesis and screening for streptomycin, amsalmycin production was increased from 1,588.4 mg/L to 2,432.2 mg/L (Yu et al., 2019). Similarly, combined ARTP mutagenesis and ribosome engineering, improved the salinomycin yield by 2.0-fold compared to that in the parental strain (Zhang et al., 2019). Therefore, by employing random mutagenesis and ribosome engineering, a strain with high butenyl-spinosyn productivity could be developed.

Indeed, the advancement of omics analysis technologies has greatly contributed to our comprehensive analysis of the high-yield mechanism (Palazzotto et al., 2019; Wang et al., 2020). For example, by employing genomics, we can sequence and analyze the entire genetic makeup of a strain, uncovering crucial genetic variations that influence the production of specific natural products (Liu et al., 2021; Khushboo et al., 2023). Transcriptomics allows us to study gene expression patterns under various conditions, shedding light on which genes are upregulated or downregulated during natural product biosynthesis (Zhang et al., 2021). Meanwhile, metabolomics provides insight into the small molecule metabolites present in a strain, allowing for the validation of intermediates associated with the biosynthesis pathway (Hasenoehrl et al., 2019). These examples validate the genuine benefits of omics analysis, which can efficiently and systematically reveal the hidden mechanisms behind antibiotic overproduction. However, there is still a lack of research in the field of omics analysis to elucidate the high-yield mechanism behind mutagenesis breeding for increased production of butenyl-spinosyn.

In this study, we first employed UV/ARTP in combination with ribosome engineering to improve the butenyl-spinosyn production. Thereafter, its high butenyl-spinosyn production performance was validated in 5 L bioreactor fermentation. To further elusive its high yield mechanism, comparative genomic, transcriptome and target metabolomic analysis were analyzed. This work aims not only to improve butenyl-spinosyn production but also to provide a proposed mechanism for understanding how various metabolic and regulatory pathways contribute to the biosynthesis of target products.

## 2 Materials and methods

### 2.1 Strains, primers, and media

All primers used in this study are summarized in Supplementary Table S1. *S. pogona* strains and their derivatives were cultivated on SP solid medium (Supplementary Table S2) at 30°C for sporulation.

### 2.2 UV/ARTP mutagenesis and streptomycin screening

Spores were cultured for 8 days for UV/ARTP mutation. For UV mutation, place 3 mL spore suspension of wild type (WT) *S. pogona* onto a sterilized plate and subsequently exposed to UV irradiation in a dark room from UV lamp with a wavelength of 254 nm and 30 W. For ARTP mutation, transfer the spore solution onto an aseptic stainless-steel plate. Then it is exposed to the plasma generated within the ARTP device (ARTP-M, Wuxi Yuanqing Tianmu Biotechnology Co., Ltd., China). To determine the minimal inhibitor concentrations (MICs) of streptomycin (Str), 1 mL 10^6^ spore suspension of *S. pogona* was placed on SP agar plates with various concentrations (0.1, 0.2, 0.3, 0.4, 0.5 mg/L) of Str and cultivated at 30°C for 7 days.

### 2.3 Fermentation and analytical methods

For butenyl-spinosyn production, spores of *S. pogona* were inoculated into 30 mL seed culture (Supplementary Table S2) in 250 mL flasks at 30°C and 220 rpm for 68–72 h. Then, 3 mL seed culture was transferred to a different fermentation medium (Supplementary Table S2) and cultivated at 30°C for 7 days. For 24-well plate cultivation, the deep well-plate is used in our work. The incubation time of 24-well plate is 5 days and the rotational speed is 220 rpm. The filling volume was 1,400 μL per well of each well-plate. The resulting filtrate was identified by HPLC (Agilent 1,100 Series, United States). Butenyl-spinosyn was separated with Agilent ZORBAX Eclipse XDE-C18 column (4.6 × 100 mm, 3.5-Micron) at 30°C using isocratic elution with a mobile phase consisting of acetonitrile: methanol: 0.05% ammonium acetate buffer at a ratio of 4.5:4.5:1, (v/v/v) with a flow rate of 1 mL/min, and detected at 254 nm. The present study utilized 5 L bioreactors (Model FUS-5 L, Shanghai Guoqiang Bioengineering Equipment Co., Ltd., China) with a fermentation medium identical to that used in the shaker culture. The cells were fermented under 30°C for 5 days. The air aeration rate used was 3 vvm. The impeller speed was installed and maintained at 350 rpm. Collect samples at intervals of 12 or 24 h to measure cellular parameters.

### 2.4 Sequencing, assembly, annotation, and analysis of genomes

The genome sequencing of mutant strain aG6 and the wild-type strain WT was conducted use of both PacBio Sequel system and Illumina NovaSeq PE150 at Novogene Bioinformatics Technology Co. Ltd. (Beijing, China). The genome sequence was assembled using SOAP, Spades, and Abyss software, and finally integrated using the CISA software. The prediction of coding sequences (CDSs) using the software Genemark. The clusters of orthologous groups of proteins (COG), gene ontology (GO), and Kyoto Encyclopedia of Genes and Genomes (KEGG) databases are used for functional annotation and pathway analysis. The software MUMmer (version 3.22) was used to detect SNPs by sequence alignment.

### 2.5 Transcriptome sequencing and comparative analysis of *S. pogona* WT and aG6

The cells were harvested from a 2 mL fermentation culture, centrifuged at 4°C, frozen with liquid nitrogen and then stored at −80°C. Majorbio Bio-pharm Technology Co., Ltd. provides sequencing and data analysis. The expression level of each gene was calculated as transcripts per million (TPM). The data point above the significance threshold (|log2 (fold change) | > 1, padj. < 0.05).

### 2.6 Sampling and quantification of intracellular metabolites concentration

In metabolomic analyses, samples are collected at 36 h and 72 h, respectively. The intracellular metabolites were extracted as described previously (Liu et al., 2019).

### 2.7 Statistical analysis

Experiments were run in three biological triplicates independently. Data were expressed as the mean values ± standard deviation. Statistical significance was analyzed by Student’s t-test (two-tail) with ***p < 0.001, **p < 0.01, and *p < 0.05, and ‘ns’ means no significance.

## 3 Results

### 3.1 Combined UV/ARTP mutagenesis and ribosome engineering for high butenyl-spinosyn producing strains

**FIGURE 1:**
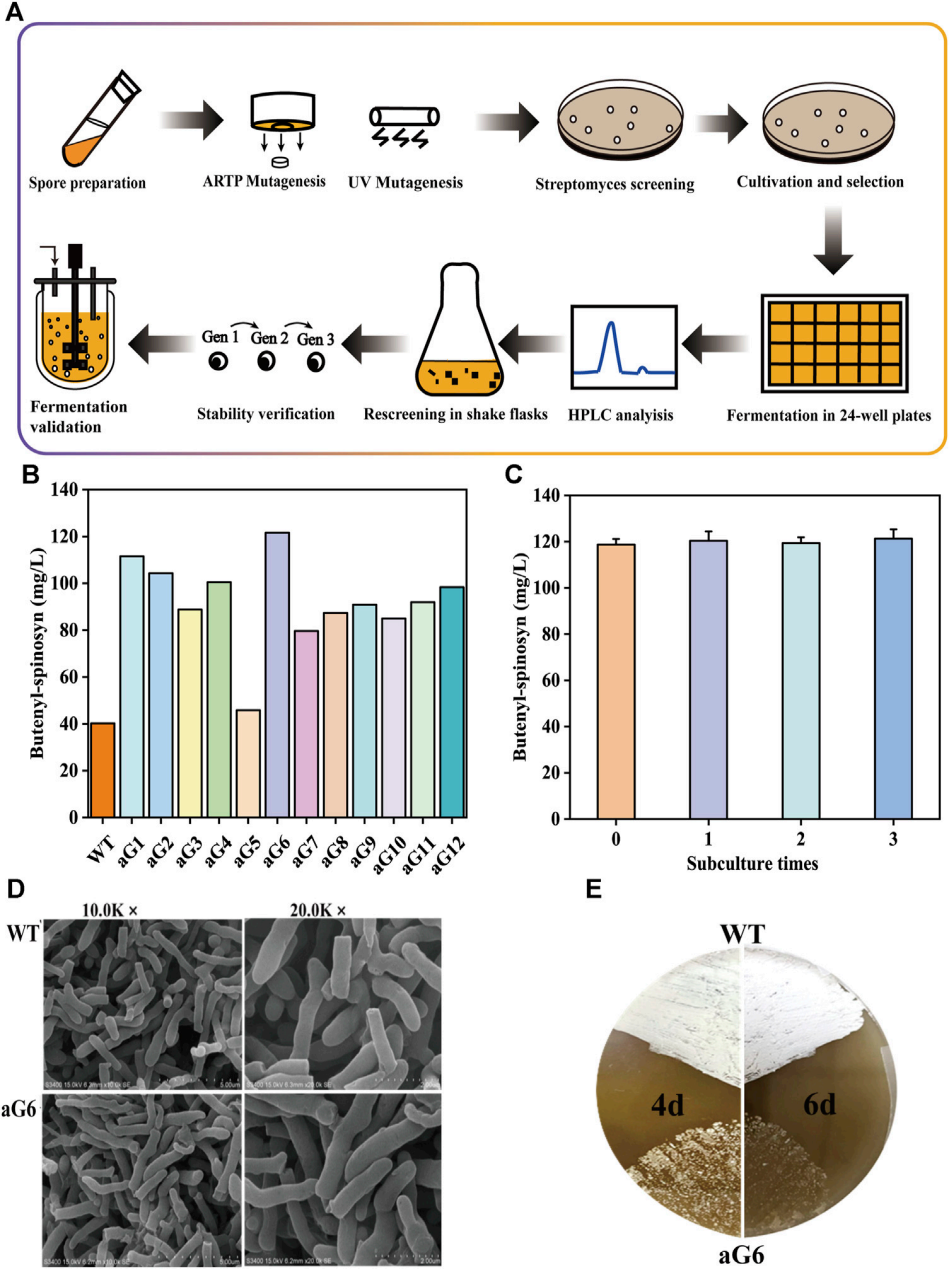
Effect of ARTP/UV combined with ribosome engineering on strain mutation. **(A)** the experimental procedure of mutant screening and validation. **(B)** rescreening of butenyl-spinosyn over-producing mutants. **(C)** production of butenyl-spinosyn in aG6 and the WT. Error bars show standard deviations. **(D)** Scanning electron morphological observation of wild-type and aG6 after 48 h. **(E)** Spores on SP agar plates grown on 7 days.

Random mutation (by UV or ARTP) along with streptomycin resistance screening methodology was used to boost butenyl-spinosyn synthesis (Figure 1A). The MIC of streptomycin against *S. pogona* was determined to be 0.3 mg/L. To determine the ARTP lethality and positive mutation rates, various treatment times (0, 32, 35, 38, 43, 45, 48, 50 s) were measured. The results showed that after 40 s of jet exposure, the spore lethality rate rose to 92%, with the greatest positive mutation rate of 11.3% recorded (Supplementary Figure S1). The lethality and positive mutation rate of UV were also determined, after 10, 20, 30, 40 s of UV irradiation. The UV treatment for 30 s resulted in 90.2% lethality and 12.4% positive mutation (Supplementary Figure S1).

The genealogical plots of *S. pogona* wild type are shown in Supplementary Figure S2. UV/ARTP-mediated mutagenesis was performed, and preliminary screening was cultured in 24-well plate. Initially, the strain was subjected to mutation using ARTP under the resistance screening of 0.3 mg/L Str. A total of 579 mutants were obtained during the ARTP process and the positive mutant rate for the wild-type strain is 32.3% (Supplementary Figure S6). As a result, the mutant strain 13-11-A5-17 was obtained, which exhibited a significant increase in butenyl-spinosyn production, reaching a titer of 95 mg/L, which is 2.4 folds higher than that of wild strain WT. Secondly, UV mutagenesis was employed for mutant breeding. This process involved subjecting the strain to UV radiation, which induced genetic mutations. Additionally, a selective pressure of 0.3 mg/L Str was applied during the breeding process. After UV mutagenesis combined with 0.3 mg/L Str screening, ZW-1 was obtained. The mutant strain ZW-1 achieved a butenyl-spinosyn production of 115 mg/L, which is approximately 45.2% higher than that of mutant strain 13-11-A5-17. To further improve the yield of butenyl-spinosyn, a concentration of 0.4 mg/L of Str was used for mutation selection. The resulting mutant, ZW-18, exhibited a butenyl-spinosyn yield of 121.6 mg/L, representing a 5.7% improvement compared to ZW-1. Then strain rescreening was performed in shaker flasks. Totally twelve mutants were cultured in shaking flasks for rescreening and were designated as aG1, aG2, aG3, aG4, aG5, aG6, aG7, aG8, aG9, aG10, aG11 and aG12, respectively (Figure 1B). Finally, a high-performance mutant name as aG6 was obtained. Additionally, the aG6 strain was confirmed to exhibit good stability in butenyl-spinosyn production (Figure 1C).

**FIGURE 2:**
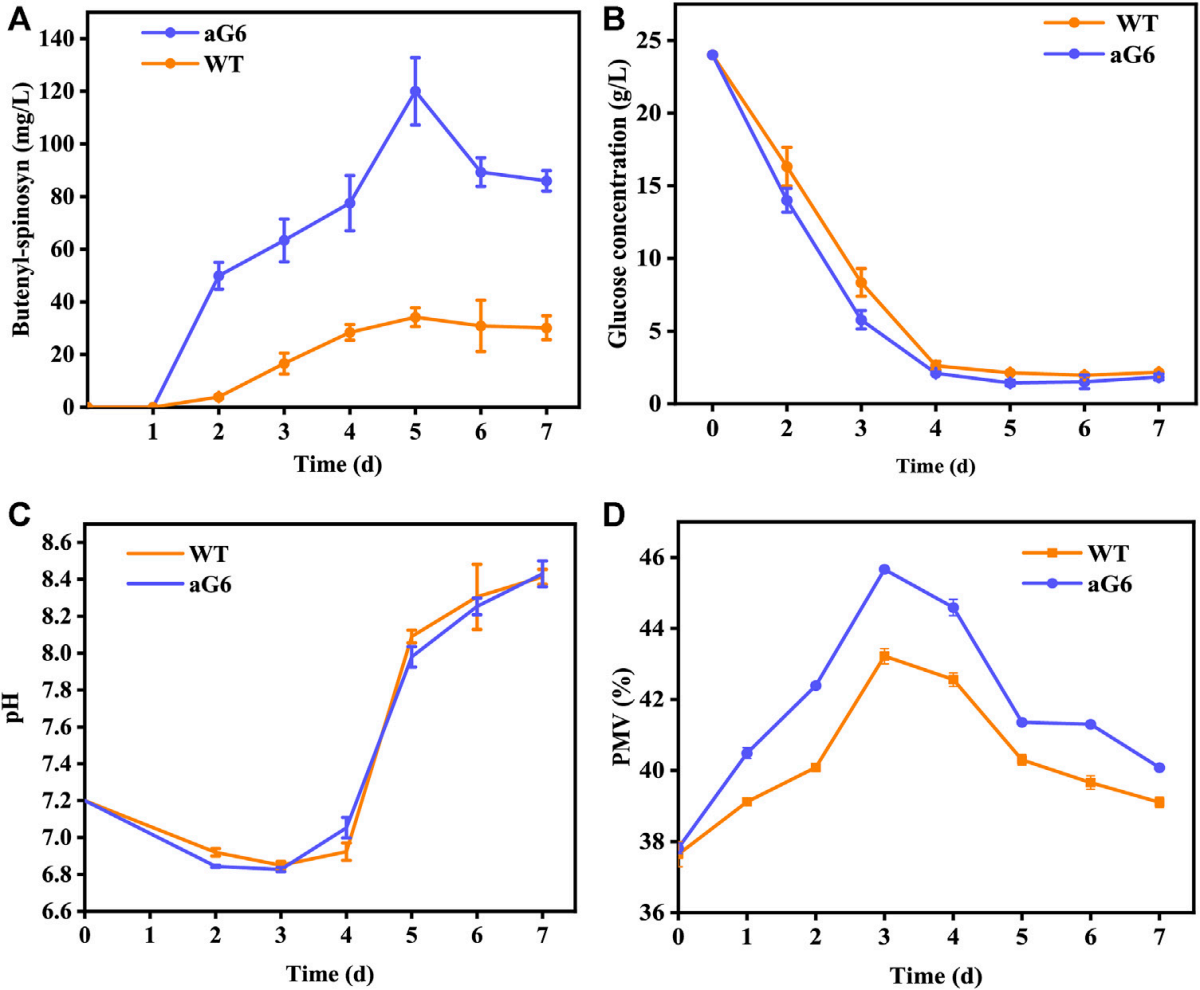
Fermentation profiles of WT and spG-1 in 250-mL shake flasks. **(A)** Time courses of residual sugar; **(B)** Butenyl-spinosyn production in WT and aG6; **(C)** pH; **(D)** cell growth (PMV).

The fermentation performance of aG6 was then examined in shaking culture. As shown in Figure 2, the maximum biomass in WT and aG6 were 46% and 43%, respectively. The glucose had quicker consumption in aG6 (Figure 2B). The time curves of butenyl-spinosyn synthesis in WT and aG6 are shown in Figure 2A. Both strains started to quickly biosynthesis butenyl-spinosyn after 2 days and reached their peak of butenyl-spinosyn production after 5 days of fermentation. Finally, the butenyl-spinosyn production by aG6 reached 122.5 mg/L in shake flask fermentation, which is 4 folds higher than that of the wild type strain. Meanwhile, we collected butenyl-spinosyns from the fermentation broth by HPLC and LC-MS analysis was used to determine the component of butenyl-spinosyn. The ingredient at 7.8 min with a m/z = 758 was found (Supplementary Figure S3), as was the ingredient at 9.4 min with a m/z = 772 (Supplementary Figure S2). This observation verified that these two components were determined as spinosyn α1 (7.8 min) and spinosyn δ1 (9.4 min), which is consistent with butenyl-spinosyn derivatives reported by Lewer et al. (2009). The difference between the two compounds is that the C6 of spinosyn α1 is connected to hydrogen, while the corresponding position of spinosyn δ1 is replaced by a methyl group. Thus, butenyl-spinosyn production of aG6 was indeed increased with UV/ARTP mutations combined with ribosome engineering.

### 3.2 Morphology differences between WT and aG6

Phenotypic comparison between WT and aG6 strains were conducted in solid and liquid medium. The WT colonies exhibit a thick appearance, while the aG6 strains produce yellowish bald spores (Figure 1E). SEM was used to observe the cell morphologies of *S. pogona* WT and butenyl-spinosyn high-producing strain aG6 (Figure 1D). We measured the thickness of the mycelium as shown in Supplementary Figure S7. The average thickness of the wild-type mycelium is 454.5 nm. The average thickness of aG6 is 695.8 nm. The results indicated that the mycelial thickness of aG6 strain was longer than that of WT *S. pogona*. To explain this phenomenon, we measured the transcription level of genes associated with hyphal morphology including *bldD*, *ssgA*, *whiB2*. The qRT-PCR analysis revealed that *bldD* significantly upregulated (Supplementary Figure S4), which could potentially be directly related to observed morphology imparity in aG6.

### 3.3 Scale-up fermentation in a 5 L bioreactor

**FIGURE 3:**
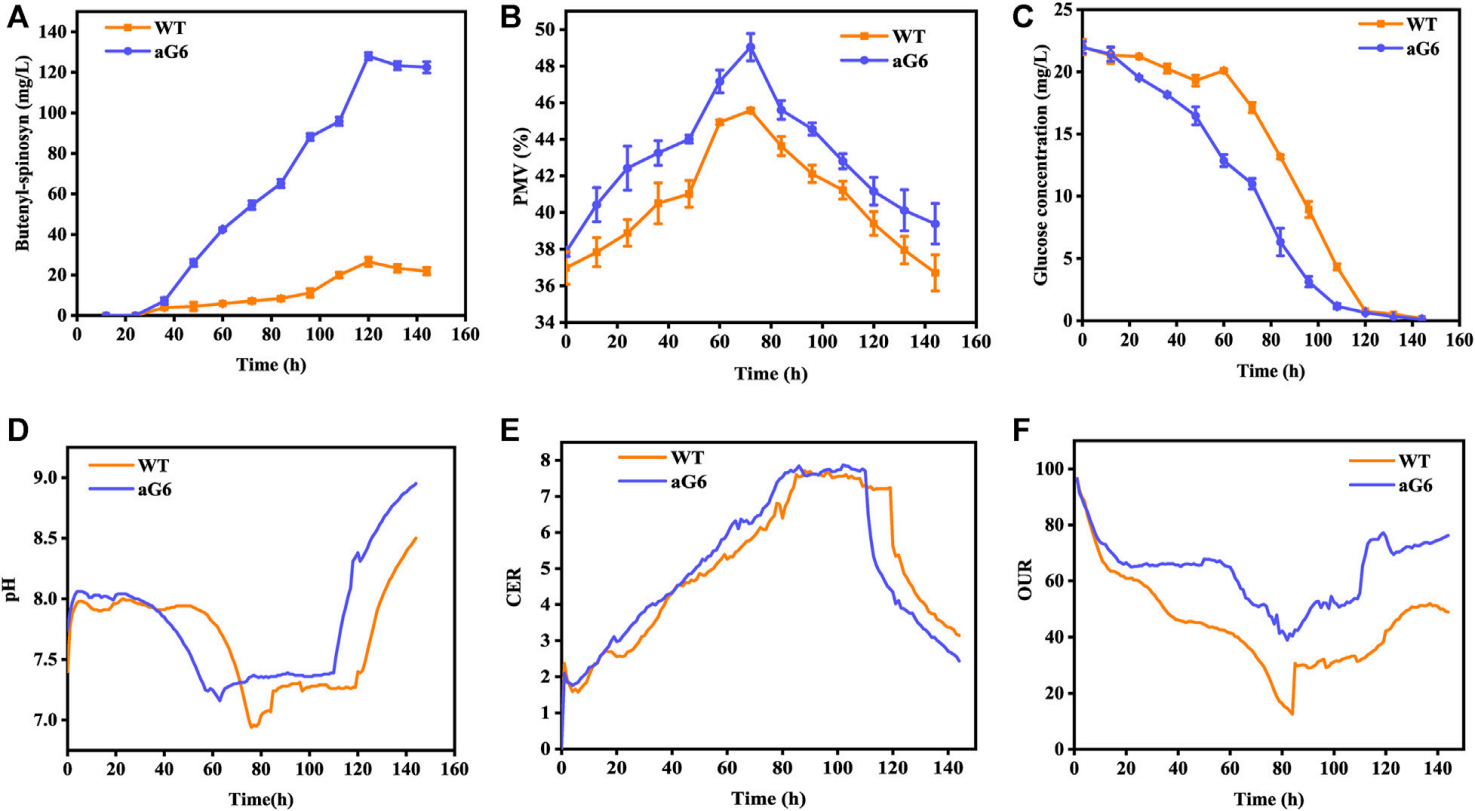
Time profiles of butenyl-spinosyn production **(A)**, PMV **(B)**, residual sugar **(C)**, pH **(D)**, CER **(E)**, OUR **(F)** in fermentation of WT and aG6 in 5 L bioreactors.

The aforementioned shake flask experiments demonstrated that the UV/ARTP mutagenesis strain aG6 produced a higher titer of butenyl-spinosyn production than the WT. To further validate their performances, bioreactor fermentations were conducted in a 5 L bioreactor (Figure 3). The physiological characteristics including changes in pH, cell growth, residual sugar, CER, OUR and butenyl-spinosyn production were examined. The carbon dioxide evolution rate (CER) and oxygen uptake rate (OUR) were higher than those of the WT (Figures 3E, F). During the butenyl-spinosyn production period after 48 h, the CER of aG6 was higher than WT and both strains exhibited CER peaks after 84 h (Figure 3E). This could be due to a higher respiratory capacity compared to WT, or it could also be attributed to an increase in biomass. As presented in Figure 3B, during the mid-stage of butenyl-spinosyn fermentation, the packed mycelium volume (PMV) continuously increases as the microorganisms utilize the nutrients in the culture medium for stable growth. However, in the late stage of fermentation, there is a slight decrease in PMV. This could be attributed to the depletion of certain nutrients in the culture medium, which imposes restrictions on the growth of strain, resulting in a decline in PMV. The PMV of strain aG6 was higher than those of the WT, indicating that both the specific growth rate and metabolic intensity of aG6 were higher (Figure 3B). During the fermentation process, the pH of the fermentation broth is constantly changing, and the pH change can reflect the cell metabolism. As shown in Figure 3D, the pH of both WT and aG6 were significantly increased at the beginning of fermentation. Due to the initial utilization of readily available nitrogen sources by the microbial cells during the early stages of fermentation, the binding of hydrogen ions causes an increase in the pH of the fermentation broth. Subsequently, a decline in pH is observed. Then pH remains stable and finally increases. However, aG6 pH drops earlier than WT because aG6 can better use carbon sources to produce organic acids, so aG6 pH preferentially decreases. The increase in pH in the later stage of growth is caused by the death of bacteria and the leakage of alkaline substances in the cell. In consideration of glucose consumption rates, aG6 showed a faster rate of glucose consumption (Figure 3C). For a producing cell, carbon source has a significant impact on the production of target products during fermentations (Jin et al., 2020). As glucose was consumed, the pH continuously decreased within 24 h (Figure 3D). Finally, the butenyl-spinosyn titer of aG6 was increased by almost 4-fold compared to WT, reaching 130 mg/L at 5th d (Figure 3A). Furthermore, it exhibits multiple-site mutations according to comparative genomic analysis of aG6 and wild-type strain WT while maintaining a high level of genetic stability. The multi-omics analysis is to explore the high-yield mechanism of butenyl-spinosyn synthesis, which has important reference value for butenyl-spinosyn production and anabolism.

### 3.4 Genomic comparison of aG6 and wild-type strain WT

**FIGURE 4:**
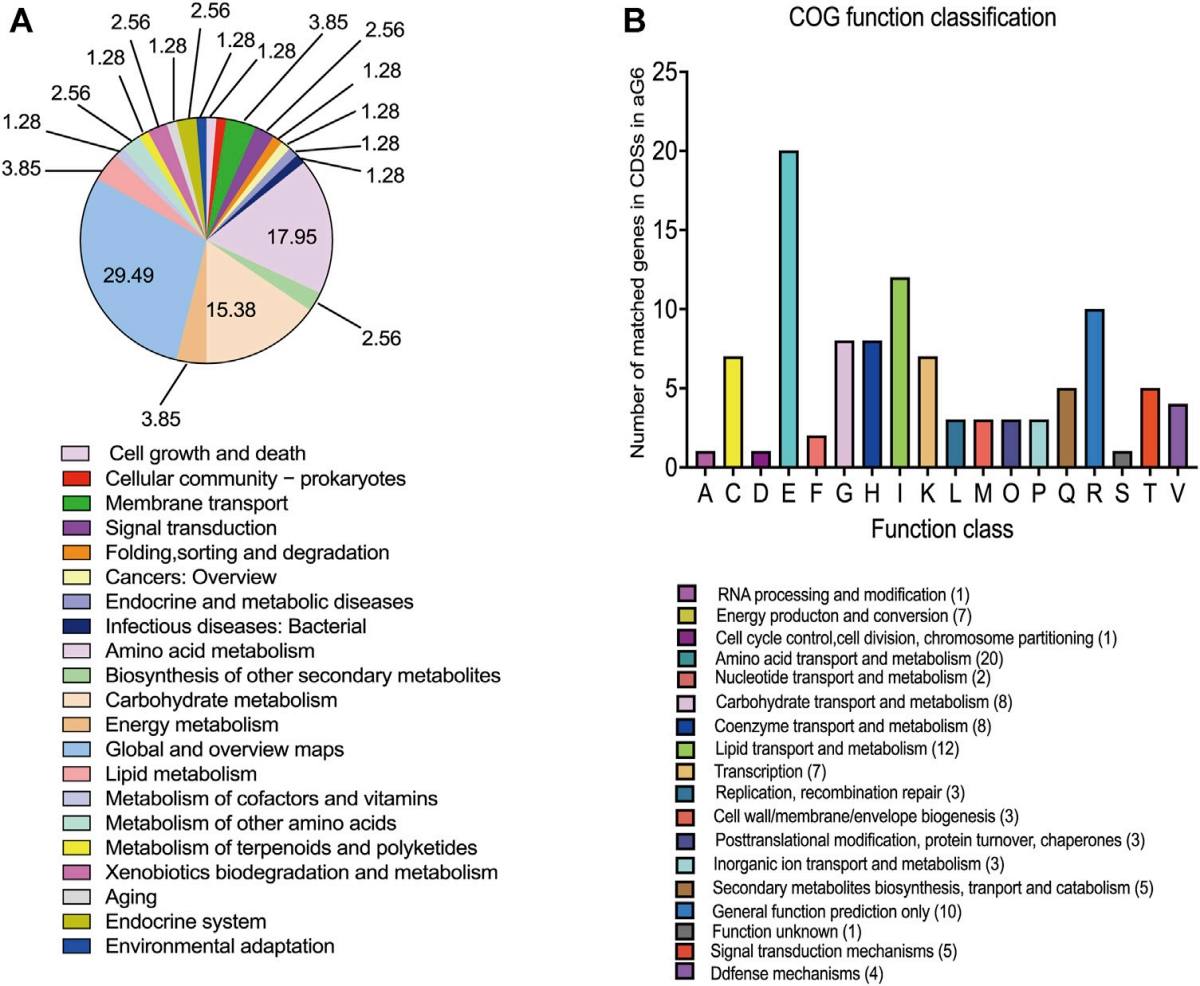
Genomic analysis of aG6 and wild-type strain WT. **(A)** KEGG pathways that were significantly enriched among the CDS mutant genes in aG6 4. **(B)** COG functional classification of aG6 CDS mutant genes.

All metabolic differences between the two strains ultimately arise from genomic mutations. To grasp a better understanding of the mechanisms behind butenyl-spinosyn overproduction, a genomic comparison was conducted between WT and the aG6. Illumina and PacBio sequencing technologies were used to sequence the whole genomes of aG6 and WT. The primary characteristics of the genome sequence of high-producing strain aG6 and wild-type strain WT are represented in Supplementary Table S3. The aG6 genome is 431.158 kb shorter than that of the wild-type strain WT. The comparison of the genomes of WT and aG6 reveals a highly conserved gene content and gene order, as well as the absence of chromosomal translocation and inversions. The aG6 exhibits an identical ncRNA profile to that of wild-type strain WT, suggesting that the translation process remains relatively conserved even after mutagenesis. The genomic changes in aG6 relative to wild-type strain WT involve 29 SNPs and 69 Indels (Supplementary Table S4). Among them, 97 mutations were identified in CDS region regions, including 17 nonsynonymous mutations that result in changes to the amino acids, potentially impacting the functionality of the corresponding proteins. It is anticipated that the coding sequences of 38 genes will be affected, including 3 regulators, 5 transposase, 24 enzymes and 6 hypothetical proteins (Supplementary Table S5). We also observed that 1 SNP and 26 Indels of aG6 were located in intergenic regions. (Supplementary Table S6). Then, we categorized the CDSs with mutations based on Kyoto Encyclopedia of Genes and Genomes (KEGG) and Clusters of Orthologous Groups (COG) protein database (Figures 4A, B). The results of KEGG pathway enrichment analysis of CDSs with mutations revealed that mutations are mostly enriched in pathways such as global and overview maps, amino acid metabolism and carbohydrate metabolism. COG analysis for CDSs with mutations showed that mutations were mostly distributed in amino acid transport and metabolism, lipid transport and metabolism, carbohydrate transport and metabolism and coenzyme transport and metabolism. There are no structural variant (SV) mutations observed between the genomes of WT and aG6. The mutations are uniformly distributed throughout the whole genome. Notably, there is a SNP mutation (S233F) in the CDS of dTDP-glucose 4,6-dehydratase (encoded by 1_orf08793) which is responsible for catalyzing the dehydration of dTDP-glucose to dTDP-4-keto-6-deoxy-glucose. The ultimate product of this pathway, dTDP-rhamnose, serves as a precursor for rhamnose. Rhamnose not only serves as a major component of the cell wall, but also plays a role in the biosynthesis of butenyl-spinosyn (Hahn et al., 2006). Meanwhile, some mutated genes encoding transposase such as 1_orf05513 (encoding IS1380 family transposase), 1_orf06188 (encoding IS110 family transposase) and 1_orf10840 (encoding IS1182 family transposase). Transposase, an enzyme capable of catalyzing transposition reactions, plays a crucial role in the movement and rearrangement of DNA or RNA sequences within and between genomes.

### 3.5 The transcriptomics analysis of aG6 and wild-type strain WT

**FIGURE 5:**
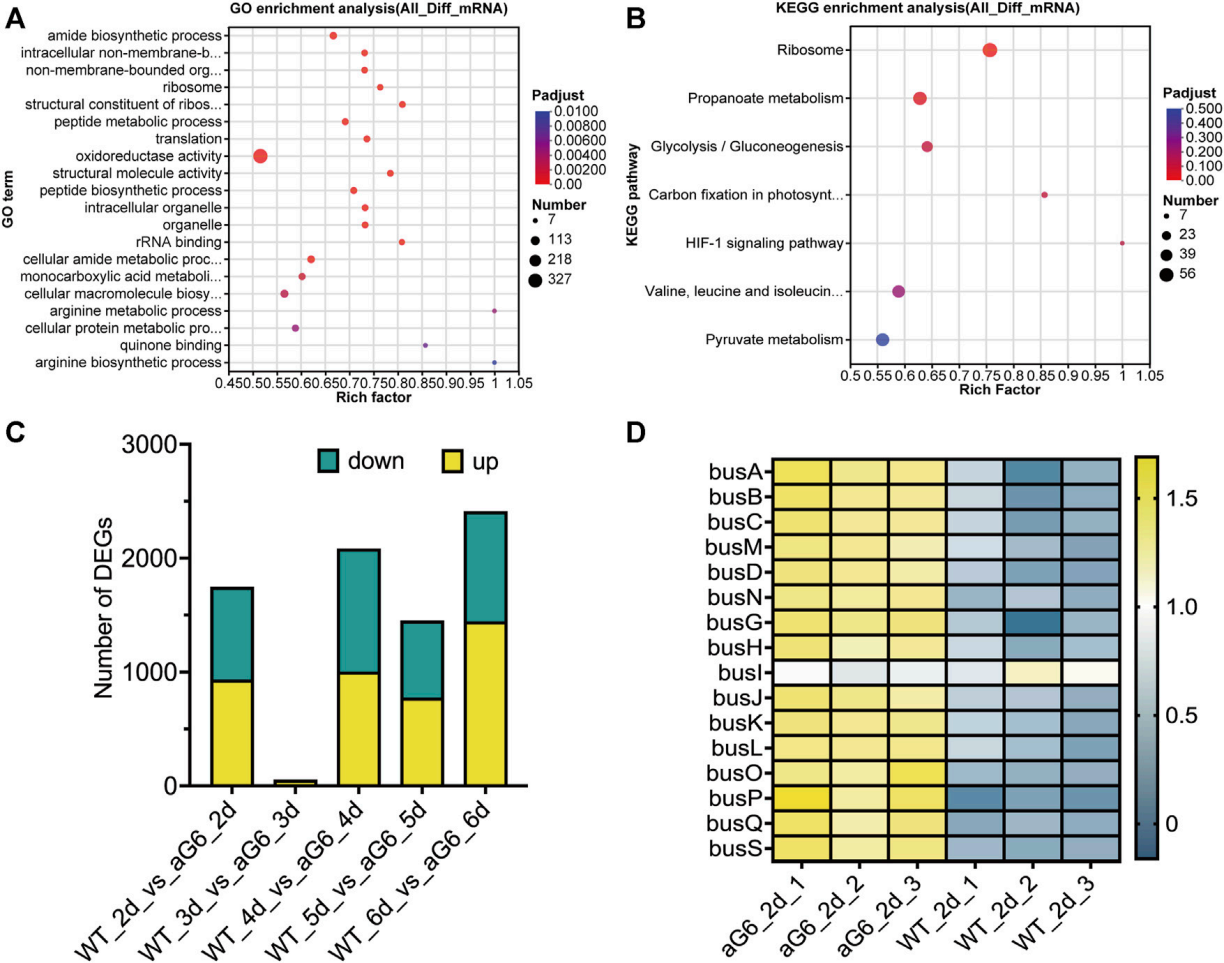
Transcriptome analysis of aG6 and wild-type strain WT. **(A)** Bubble plot of the most significant pathways with KEGG enrichment of DEGs. **(B)** Bubble plot of the most significant pathways with GO enrichment of DEGs. **(C)** Comparative transcriptome analysis of the DEGs between strains aG6 and wild-type strain WT after cultivation for 2, 3, 4, 5 and 6 days. **(D)** Hierarchical clustering of genes in butenyl-spinosyn biosynthesis gene cluster at day 2.

The differences among high-yield mutant strain aG6 and wild-type strain WT transcriptomes were analyzed using total RNAs collected after cultivation for 2, 3, 4, 5 and 6 days. Differentially expressed genes (DEGs) were mainly focused on the 3 and 6 days. The expression of 2,406 genes exhibited differences between aG6 and WT at day 3, with 1,439 had an upregulation and 967 had a downregulation, as 999 and 1,080 genes were upregulated and downregulated at day 6 (Figure 5C). To obtain an overview of the biological function of DEGs, GO and KEGG annotation were respectively shown in Figures 5A, B. The results of GO analysis for DEGs revealed that DEGs are mostly enriched in pathways such as amide biosynthetic process, intracellular non-membrane-bounded organelle, non-membrane-bounded organelle, ribosome and oxidoreductase activity. KEGG pathway enrichment analysis of DEGs revealed that the most affected pathways were a) Ribosome, b) Propanoate metabolism, c) Glycolysis, d) Carbon fixation in photosynthetic organisms, and e) HIF-1 signaling pathway.

#### 3.5.1 DEGs related to secondary metabolite synthesis

The synthetic gene cluster of butenyl-spinosyn was identified, and transcriptomic analysis revealed that the expression of genes related to butenyl-spinosyn biosynthesis gene cluster was drastically upregulated in aG6 at day 2 (Figure 5D). Moreover, the transcription levels of butenyl-spinosyn biosynthesis genes as shown by the RNA-seq data were largely consistent with those determined by qRT-PCR (Supplementary Figure S5). This consistency indicates the accuracy of the RNA-seq results.

#### 3.5.2 Putative mechanism of ARTP/UV mutagenesis and ribosomal engineering to improve butenyl-spinosyn production

**FIGURE 6:**
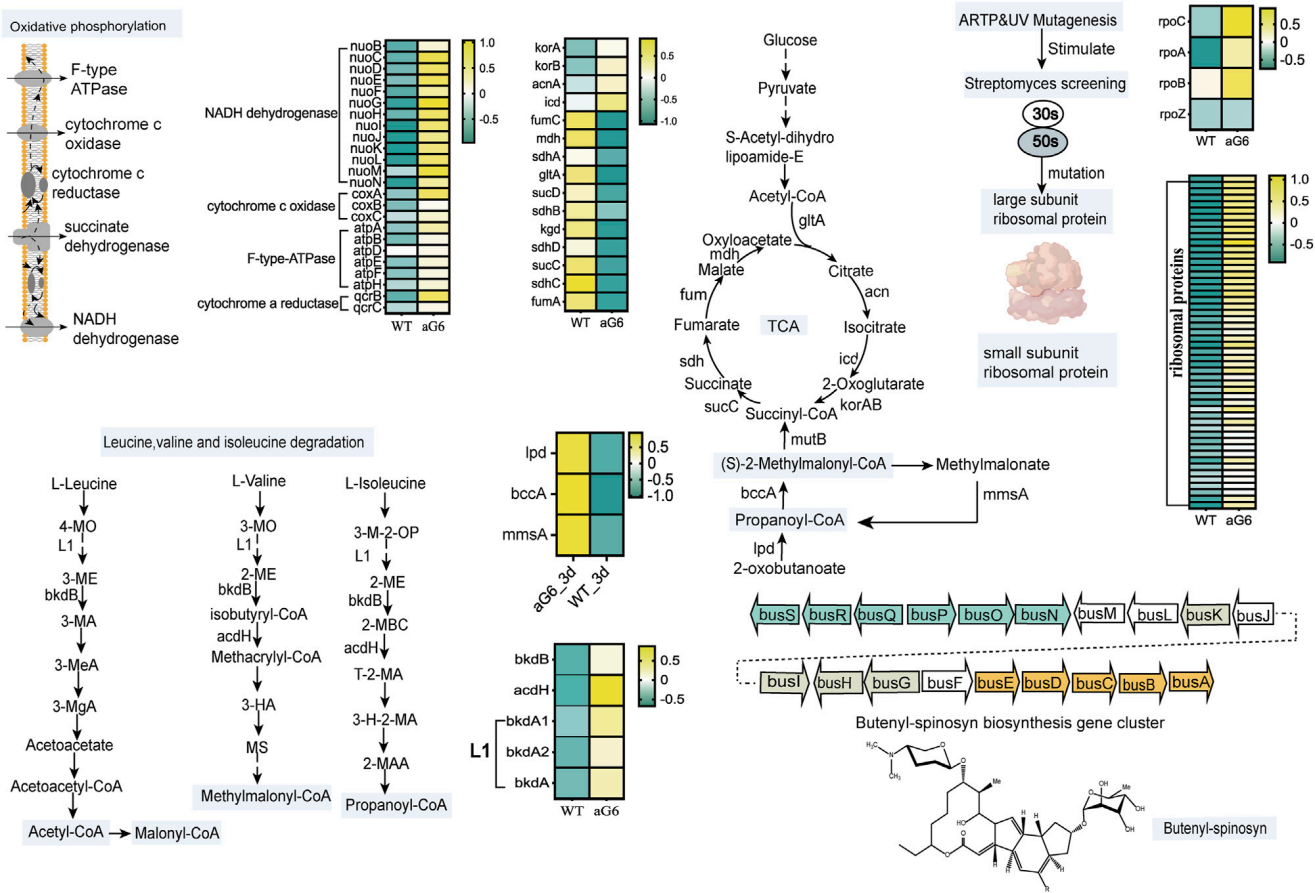
Proposed mechanism of ARTP/UV mutagenesis and resistance screening to improve butenyl-spinosyn production. Heatmap colors indicate the gene expression level (Log2FC) in each sample as shown on the right.

After scrutinizing the pathways associated with butenyl-spinosyn biosynthesis, an initial regulatory model of high production of *Saccharopolyspora pogona* aG6 strain after UV/ARTP mutagenesis and ribosomal engineering is proposed (Figure 6). In general, ARTP/UV mutagenesis could induce breakage in DNA and oligonucleotides such as insertion, deletion and missense mutation (Zhuang et al., 2019). Zhu et al. found that ribosome engineering technology targeting ribosomal proteins and some translation factors, eventually activates the production of antibiotics (Zhu et al., 2017). Ribosomes are essential cellular components responsible for synthesizing proteins. The component of ribosomes is ribosomal ribonucleic acid (rRNA), and it has a large and small subunit. The large subunit plays indispensable role in polypeptide biosynthesis, while the small subunit is responsible for mRNA decoding (Liu et al., 2023). Therefore, we first focused on the ribosomal proteins, and found that a series of genes related to the expression of ribosomal proteins were significantly upregulated at day 6 (Figure 6; Supplementary Table S7). Moreover, ten genes involved in expression of ribosomal proteins were significantly upregulated at day 3 (Supplementary Table S8). Particularly, a previous study has indicated that S12 participates in the process of identifying codon and anticodon pairs, and in the subsequent conformational rearrangements of the 30S subunit (Agarwal et al., 2011). RNA polymerase plays a critical role in gene expression, as it allows the genetic information encoded within DNA to be converted into functional RNA molecules (Sandoz et al., 2023). The transcription of RNA polymerase rpoC (1_orf04248), rpoA (1_orf04149), rpoB (1_orf04250) increased as these were overexpressed in aG6 strain (Figure 6; Supplementary Table S7). These results suggest that the utilization of ARTP mutagenesis and resistance screening can generate mutant strains that display genetic mutations in ribosomal proteins and RNA polymerase.

As it is well known, biosynthetic precursors play a pivotal role in secondary metabolite productivity (Li et al., 2021). Malonyl-CoA (M-CoAs), methylmalonyl-CoA (MM-CoAs), and propionyl-CoA are indeed considered as the essential precursors in the biosynthesis of butenyl-spinosyn (An et al., 2021). Thus, focusing on the central carbon metabolism and genes involved in precursors synthesis pathway is a rational approach to study the biosynthesis of butenyl-spinosyn. Firstly, we found that genes participate in propanoate metabolism pathway, including 1_orf02121 (*lpd*) coding for dihydrolipoamide dehydrogenase, 1_orf04537 (*bccA*) coding for propionyl-CoA carboxylase, 1_orf08042 (*mmsA*) coding for malonate-semialdehyde dehydrogenase were upregulated at day 3. Increased transcription levels of propanoate metabolism pathway may favor the accumulation of propionyl-CoA and MM-CoAs, thereby promoting the butenyl-spinosyn biosynthesis. (Guo et al., 2022).

As acetyl-CoA enters the tricarboxylic acid cycle (TCA), the aconitate hydratase gene 1_orf12664, responsible for catalyzing the conversion of citrate to isocitrate, was significantly upregulated at day 6 in aG6 (Figure 6; Supplementary Table S7). Furthermore, the expression of isocitrate dehydrogenase (1_orf03853) in the pathway from isocitrate to 2-oxoglutarate was upregulated. Additionally, the expression of two genes (1_orf00954 and 1_orf00953) encoding components of 2-oxoglutarate dehydrogenase, which catalyze the conversion of 2-oxoglutarate to succinyl-CoA, was also increased at day 3 and day 6 (Supplementary Tables S7, S8). Besides, the transcription level of the three-step reaction from succinyl-CoA to fumarase was significantly downregulated in aG6 (Figure 6; Supplementary Table S7). Succinyl-CoA mainly flows in two directions: one is to participate in the TCA cycle to synthesize succinate, and the other is to convert into methylmalonyl-CoA for the synthesis of precursors for butenyl-spinosyn. Therefore, this could potentially facilitate the supply of precursors for the biosynthesis of butenyl-spinosyn.

In aG6, for the leucine, valine, and isoleucine degradation pathways, the expression of gene 1_orf04535, encoding acyl-CoA dehydrogenase was significantly upregulated at day 3 and day 6 (Supplementary Tables S7, S8). Moreover, four genes (1_orf05171,1_orf05176,1_orf05175 and 1_orf00879) were significantly upregulated at day 6 (Figure 6; Supplementary Table S7). Amino acids are not only important building blocks for protein synthesis but also serve as precursors for various metabolic pathways, including the synthesis of polyketides. The branched chain amino acids (leucine, valine, and isoleucine) degradation is one of the main metabolic pathways that provide precursors of polyketides synthesis. Prior research revealed that overexpression associated with branching amino acids of three factors/operating ilvB1, bdkOp and mmsOp1 all resulted in increased erythromycin yields (Karnicar et al., 2016). We speculated that ARTP/UV treatment and resistance screening have resulted in an increased anabolic flux through butenyl-spinosyn precursors synthesis pathway in aG6.

Oxidative phosphorylation is an important energy metabolism process. Oxidative phosphorylation occurs on electron transport chains that are organized into four membrane-embedded oligomeric assemblies of proteins, including NADH dehydrogenase, succinate dehydrogenase, CoQ-cyt c oxidoreductase and cytochrome c oxidase (Bu et al., 2021). Oxidative phosphorylation plays a crucial role in the synthesis of antibiotics. Primary metabolism is closely linked to secondary metabolism by providing specific precursors and cofactors (Li et al., 2020). The biosynthesis process of butenyl-spinosyn is regulated by complex secondary metabolism, and is closely related to primary metabolism because of the supply of precursors and cofactors, for example, NADH is directly required as a reducing force during its synthesis. According to the comparative transcriptomics analysis, the expression of genes encoding key enzymes of oxidative phosphorylation was also significantly upregulated in turn affecting the flux of metabolites through butenyl-spinosyn feeder pathways (Figure 6; Supplementary Table S7). The upregulation of oxidative phosphorylation promotes energy production. The biosynthesis of butenyl-spinosyn is an energy-intensive process. In other words, relevant genes' upregulation could boost energy levels and subsequently promote butenyl-spinosyn biosynthesis.

### 3.6 Comparison of the intracellular metabolomics in aG6 and wild-type strain WT

The intracellular amino acid and phosphate sugar from the aG6 and wild-type strain WT at 2 cultivation time points (36 h and 72 h) were obtained and analyzed by isotope-assisted GC-IDMS. The intracellular coenzyme A from the aG6 and wild-type strain WT at 36 h were obtained and analyzed by UPLC–MS/MS. A total of 27 intracellular metabolites were identified and quantified by using standards, encompassing amino acids, phosphate sugars and coenzyme A from the central carbon metabolism. These metabolites assume crucial functions in diverse cellular processes, serving as pivotal intermediates in both energy production and biosynthesis.

#### 3.6.1 Amino acid analysis

**FIGURE 7:**
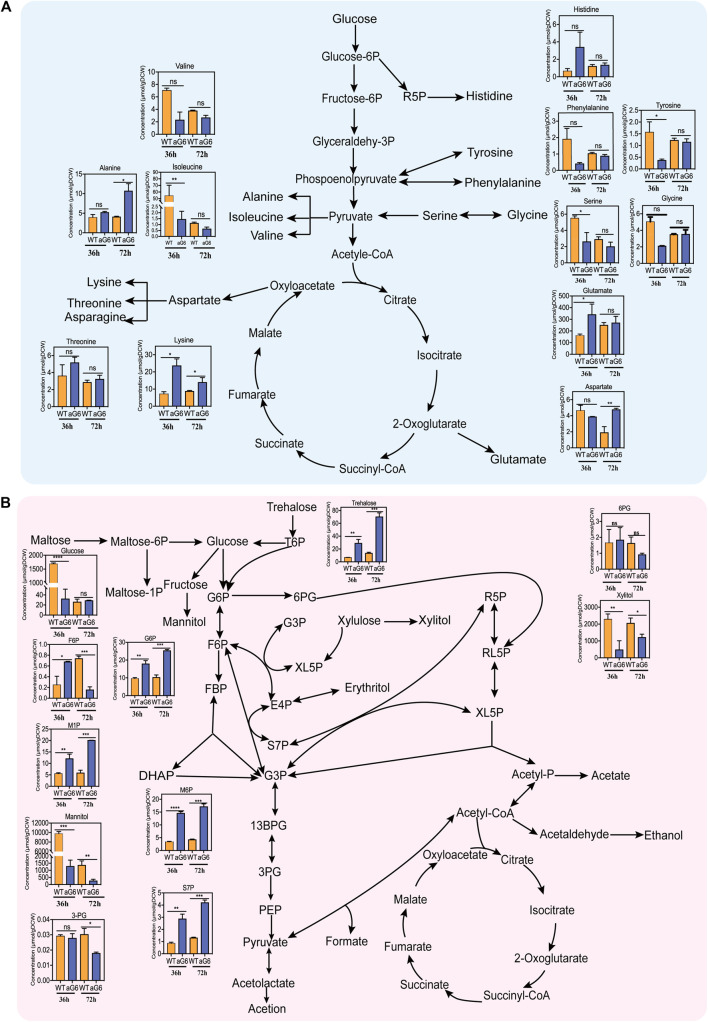
Intracellular metabolites network of *S. pogona.* Amino acids in *S. pogona* WT and aG6 at 36 h and 72 h **(A)**. Phosphate sugar and organic acids in *S. pogona* WT and aG6 at 36 h and 72 h **(B)**.

Amino acids play a crucial role in supporting cell growth and serving as precursors for polyketide synthesis. The amino acids pool sizes for aG6 and wild-type strain WT at 36 h and 72 h are shown in Figure 7A. At 36 h, there was a significant decrease in the contents of both isoleucine and valine (Figure 7A). Valine, and isoleucine are members of the pyruvate (Pyr) family which are produced from pyruvate via the EMP pathway (Yuan et al., 2023). Surprisingly, both isoleucine and valine showed a similar downward trend, with isoleucine exhibiting a significant decrease at two different time points. Isoleucine serves as a direct precursor for the biosynthesis of propionyl-CoA. Thus, it is possible that the amino acids from the Pyruvate (Pyr) family might serve a similar function in supplying precursors for butenyl-spinosyn biosynthesis. The decrease in the levels of these two amino acids is consistent with an increase in their degradation pathways, as observed in the transcriptome data. Notably, the content of lysine showed a significant increase at both 36 and 72 h. According to KEGG metabolic pathway analysis, TCA cycle, glycolysis and pyruvate metabolism are involved in lysine biosynthesis. The aforementioned comparative transcriptome data analysis revealed that some genes participating in TCA cycle, glycolysis and pyruvate metabolism showed an upregulation pattern in aG6. Therefore, we speculated that the accumulation of lysine may be beneficial for central carbon metabolism in aG6. Additionally, the pool size of glutamate in aG6 was distinctively larger than in WT at 36 h. Notably, glutamate could be converted to succinyl-CoA, which can be isomerized by methylmalonyl-CoA mutase (MCM) to synthesis methylmalonyl-CoA (Liu et al., 2018), another major elongation unit of butenyl-spinosyn biosynthesis. Thus, it could be inferred that an increase in the glutamate pool size potentially facilitates more methylmalonyl-CoA towards the biosynthesis of butenyl-spinosyn. Likewise, the significant accumulation of amino acids ensured the supply of building blocks for cell growth and subsequent butenyl-spinosyn synthesis.

#### 3.6.2 Sugar phosphate analysis

Phosphate sugars are intermediate products of carbon metabolism that are crucial for multiple biochemical pathways at the cellular level. The sugar phosphate pool sizes of aG6 and wild-type strain WT at 36 h and 72 h are shown in Figure 7B. In aG6, there was an increase in the pool size of glucose-6-phosphate (G6P) and fructose-6-phosphate (F6P) at 36 h. Both G6P and F6P are intermediate metabolites in the EMP pathway. G6P, in particular, serves as a key node in various sugar metabolism pathways. The significant increase of the G6P and F6P in aG6 ensures a sufficient precursor supply for various sugar metabolism pathways. On the other hand, a larger pool size of S7P was observed in aG6 at 36 h and 72 h. S7P serves as an intermediate in the pentose phosphate pathway. The PP pathway not only provides necessary precursors for the growth of the strain but also plays a crucial role in providing the reducing power NADPH for protein synthesis (Lu et al., 2015). The PP pathway is essential for both the production of biomass and the synthesis of proteins due to its role in generating NADPH. Thus, we hypothesize that the increased S7P content facilitates the supply of precursors and NADPH required during butenyl-spinosyn biosynthesis. Additionally, the levels of trehalose in aG6 were significantly higher than in WT at 36 h and 72 h. Trehalose has the function of protecting bacteria from environmental stresses and reducing oxidative stress caused by oxygen in bacteria. The high content of trehalose might enhance the cellular antioxidant capacity.

#### 3.6.3 Coenzyme A analysis

A total of four coenzymes including propionyl-CoA, aceyl-CoA, succinyl-CoA and malonyl-CoA were identified between aG6 and wild-type strain WT. The results revealed that propionyl-CoA and succinyl-CoA were significantly increased in aG6 (Supplementary Figure S8). The conversion of succinyl-CoA to methylmalonyl-CoA can be catalyzed by methylmalonyl-CoA mutase and methylmalonyl-CoA can be further converted to propionyl-CoA. Propionyl-CoA can be incorporated into polyketides as a starter unit directly (Li et al., 2021). Specifically, succinyl-CoA is used to synthesise succinate through succinyl-CoA synthetase in the TCA. It has been reported that the insertion of inactivated succinyl-CoA synthetase can divert more carbon sources towards erythromycin, resulting in an increase in erythromycin production (Ke et al., 2022). Therefore, a significant increase in succinyl-CoA and propionyl-CoA content may favor the supply of precursors for the synthesis of butenyl-spinosyn.

## 4 Discussion

Despite remarkable progress in increasing butenyl-spinosyn production through various attempts (Table 1), its relatively low titer renders it unsuitable for applications. The combination of mutagenesis is a viable and efficient approach, capable of rapidly enhancing the titer of strains. For instance, by subjecting *Staphylococcus aureus* ATCC 14891 to titanium sapphire laser and oxalic acid screening, high-yielding strain FS35 was obtained, with FK520 production reaching 305.6 mg/L (Qi et al., 2012). Chen et al. reported that utilization of ARTP in conjunction with sodium nitrite resulted in a 26.9% increase in the titer of sophorolipids compared to the original *C. bombicola* strain (Chen et al., 2020). This research is the first attempt to combine random mutagenesis and ribosome engineering to improve butenyl-spinosyn production. In addition, the fermentation validation in 5 L bioreactor facilitates our precise monitoring of critical process parameters and laying the foundation for scale-up production of butenyl-spinosyn.

**TABLE 1:** Methods of improving the yield of butenyl-spinosyn.

Technique	Methods	Improvement in butenyl-spinosyn production	References
Random mutagenesis	NTG mutagenesis	86.7%	Chen et al. (2018)
	Ribosome engineering	1.79-fold	Luo et al. (2016)
	Ribosome engineering	2.2-fold	Wu et al. (2015)
Metabolic engineering	Flaviolin-like gene cluster deletion	4.06-fold	He et al. (2021b)
	Overexpression Sp1418	225.9%	He et al. (2020)
	Combined modification of butenyl-spinosyn polyketide synthase and succinic semialdehyde dehydrogenase	154.1 ± 10.98 mg/L	Rang et al. (2022)
	NRPS-T1PKS cluster deletion	4.72-fold	Rang et al. (2021)
	Overexpressed polynucleotide phosphorylase gene	1.92-fold	Li et al. (2018)

Random mutagenesis has been widely employed in the mutagenesis of industrial strains such as fungi, bacteria, yeast, and actinomycetes. This method significantly improves the synthesis efficiency of target products. However, there is limited research on the high-yield mechanisms that underlie the increased production of desired products in mutagenesis breeding. Omics analysis has proven invaluable in elucidating the intricacies of intracellular metabolism and regulatory networks in microbial strains. Li et al. further improve the production of erythromycin in mutagenesis-derived high-yield strains through comprehensive omics analysis (Li et al., 2022). Wang et al. employed a multi-omics analysis to uncover the intracellular triacylglycerol (TAG) metabolic regulation (Wang et al., 2020). In this study, we first time present a mechanism for enhancing butenyl-spinosyn production in mutagenesis breeding based on multi-omics analysis. Given the modest progress in enhancing butenyl-spinosyn production, we hold the belief that the utilization of omics analysis will prove to be of significance in future endeavours pertaining to strain. Through comparative genomic analysis, we have discovered that the genes undergoing mutations are primarily concentrated within pathways such as amino acid metabolism and carbohydrate metabolism, lipid transport and metabolism and coenzyme transport and metabolism. Particularly, we noted a SNP occurs in CDS of dTDP-glucose 4,6-dehydratase. It is reported that through the increase in the supply of TDP-4-keto-6-deoxy-D-glucose could improve spinosyn production (Pan et al., 2011). Hence, it is worthwhile to verify this mutation target in future work. By conducting a comparative transcriptomic analysis, we proposed that the alterations of ribosomal proteins, tricarboxylic acid cycle, branched-chain amino acid degradation and oxidative phosphorylation could be the main reasons for the higher production of butenyl-spinosyn. Although some DEGs are clearly associated with butenyl-spinosyn high-yield, there are still other observed differences between WT and aG6 strains that lack a comprehensive analysis and rational explanation. Metabolomic analysis of WT and aG6 revealed that fluctuations in the levels of intracellular metabolites potentially entwined with the synthesis of precursors essential for butenyl-spinosyn. This study can not only help researchers study in bioprocess engineering of the butenyl-spinosyn, but also provide useful clues to help achieving the goal of improving the production of butenyl-spinosyn. Nevertheless, the interrelationships among genomic, transcriptome and target metabolomic remain enigmatic. Therefore, future research should focus on the integration analysis of multi-omics to elucidate the high-production mechanisms of butenyl-spinosyn with greater clarity.

## Data Availability

The datasets presented in this study can be found in online repositories. The names of the repository/repositories and accession number(s) can be found below: The raw datasets of time-course transcriptome and comparative transcriptome between aG6 and wild- type used in this study have been uploaded to NCBI (NCBI BioProject Accession: PRJNA1007868). The genome sequence of *S. pogona* high-yield strain aG6 were deposited in NCBI BioProject database under the accession number PRJNA1032256.
