# Supplementary material for: Mechanistic insight for improving butenyl-spinosyn production through combined ARTP/UV mutagenesis and ribosome engineering in Saccharopolyspora pogona
Source: Front Bioeng Biotechnol. 2024 Jan 15;11:1329859. doi: 10.3389/fbioe.2023.1329859 (PMC10825966; doi:10.3389/fbioe.2023.1329859)
Supplement: Supplementary file 1 [file DataSheet1.docx]

**Supplementary information**


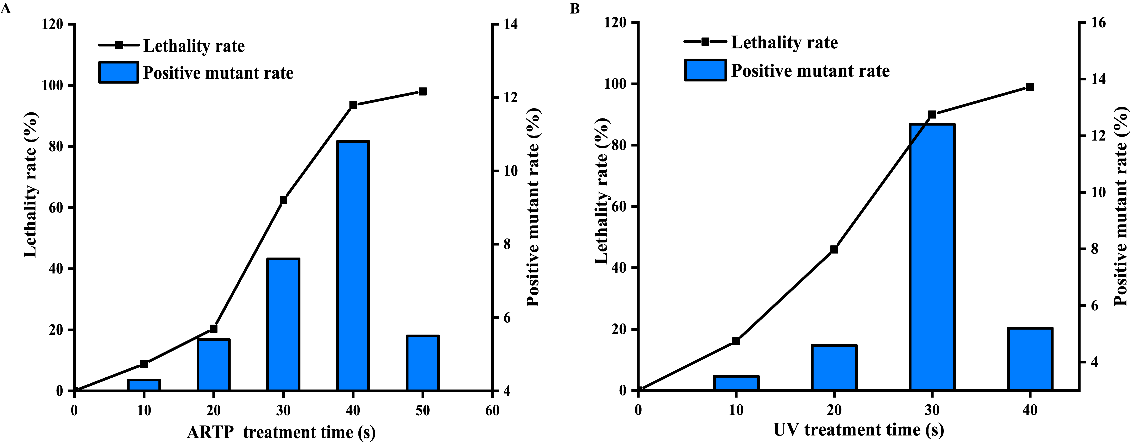


**Fig. S1**. Lethality rate and mutation rate of *S. pogona* WT. (A) Lethality rate and mutation rate of *S. pogona* WT by ARTP mutagenesis. (B) Lethality rate and mutation rate of *S. pogona* WT by UV mutagenesis.


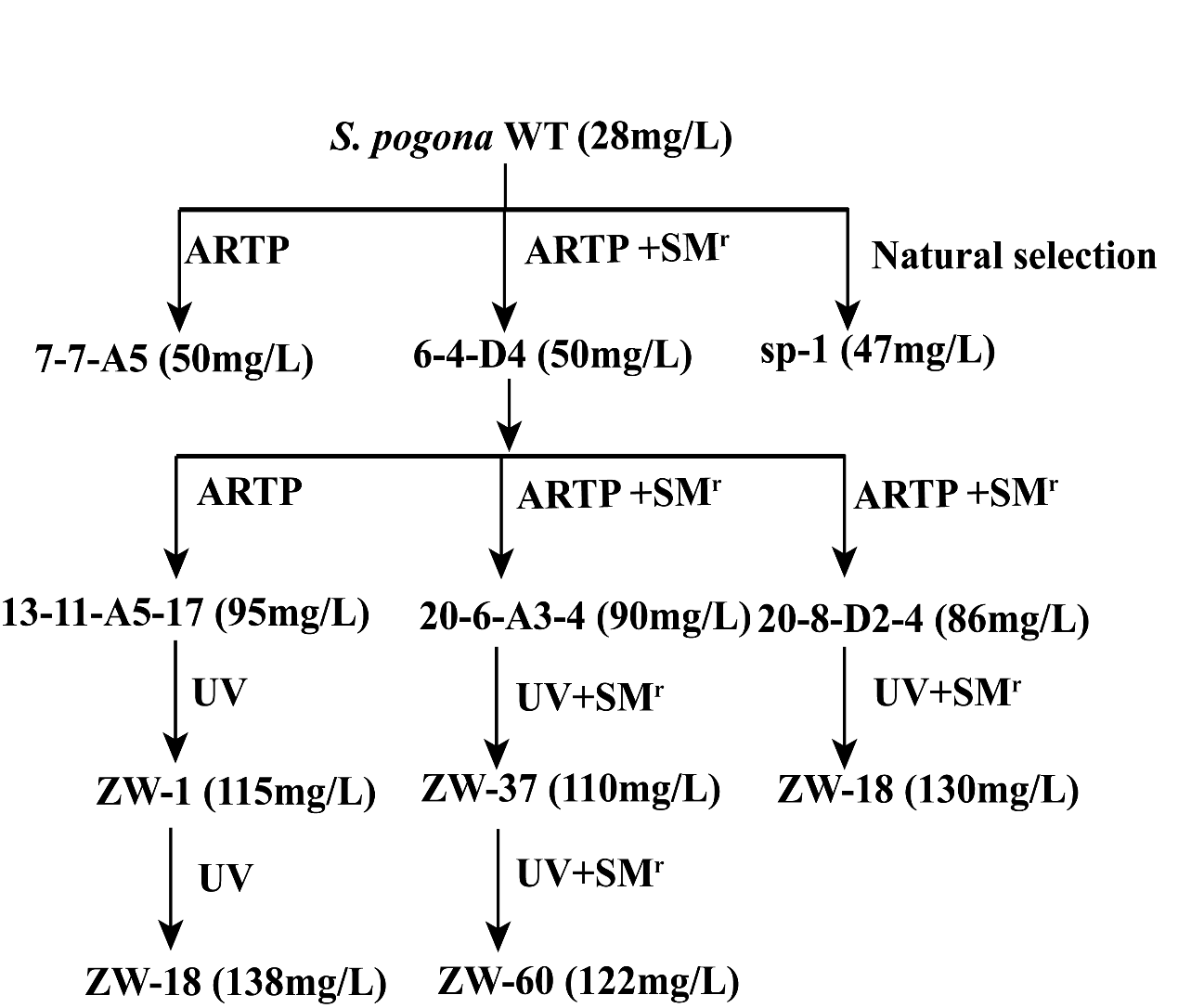


**Fig. S2**. Tree of *S. pogona* WT with UV/ARTP radiation and streptomycin resistance screening model. SMr*, 0.3 mg/L streptomycin; SMr**, 0.4 mg/L streptomycin.


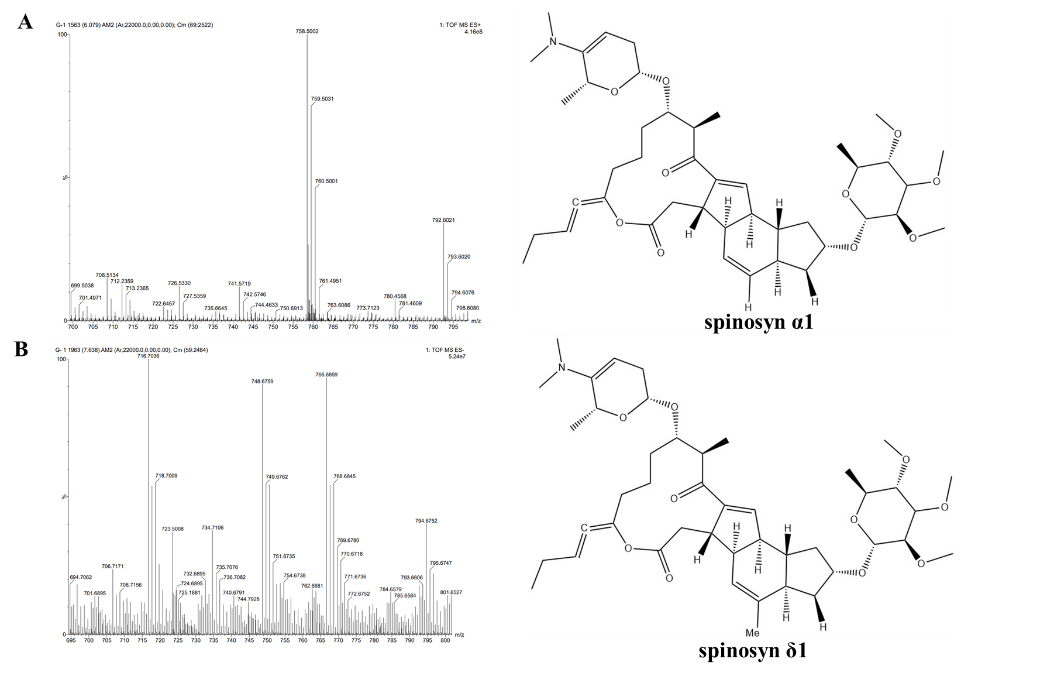


**Fig. S3.** The substances were identified by LC-MS. (A) MS identification results showed that m/z =758, which was confirmed as a butenyl-spinosyn component spinosyn α1. (B) MS identification results showed that m/z =772, which was confirmed as a butenyl-spinosyn component spinosyn δ1.


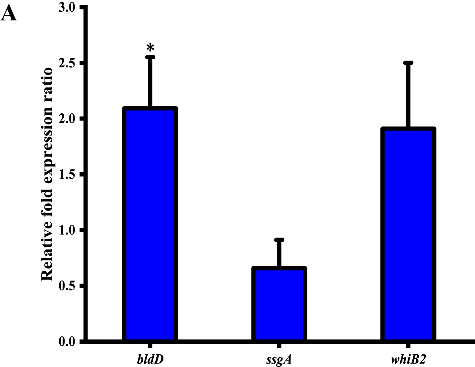


**Fig. S4.** Relative expression levels of *bldD*, *ssgA* and *whiB2* in *S. pogona* WT and aG6.


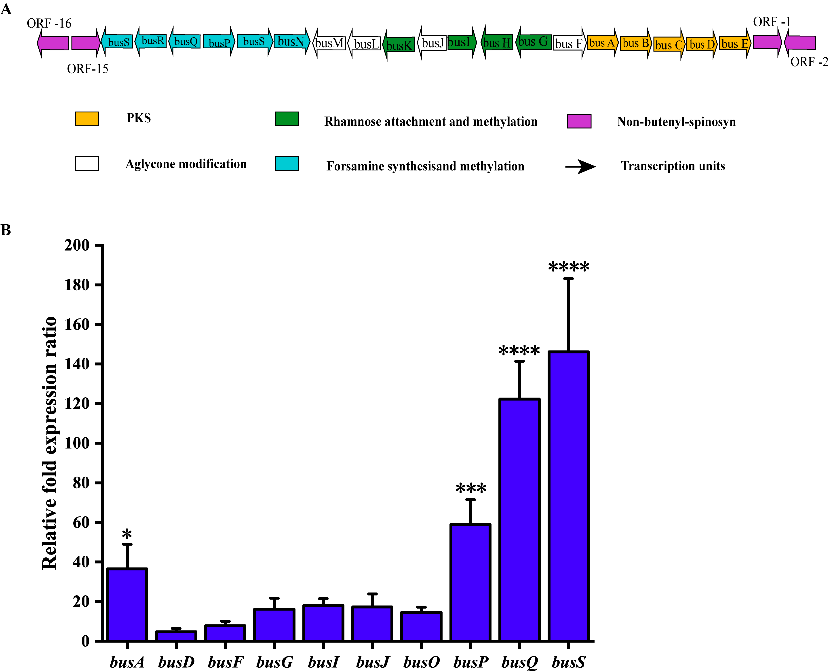


**Fig. S5.** The butenyl-spinosyn biosynthetic genes are located in different transcription units in *S. pogona*. (A) Each black arrow represented the genes located in the same transcription units. (B) Relative expression level of butenyl-spinosyn biosynthetic gene cluster in *S. pogona* aG6, compared with those of WT. In total, ten genes were chosen for indicating the expression level of co-transcription units. Fold change was normalized against the mean value of WT ,***P < 0.001.


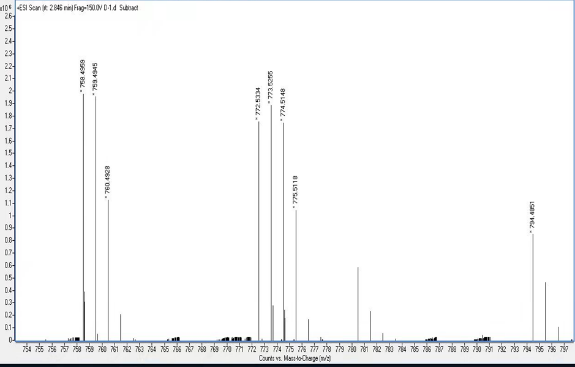


**d**


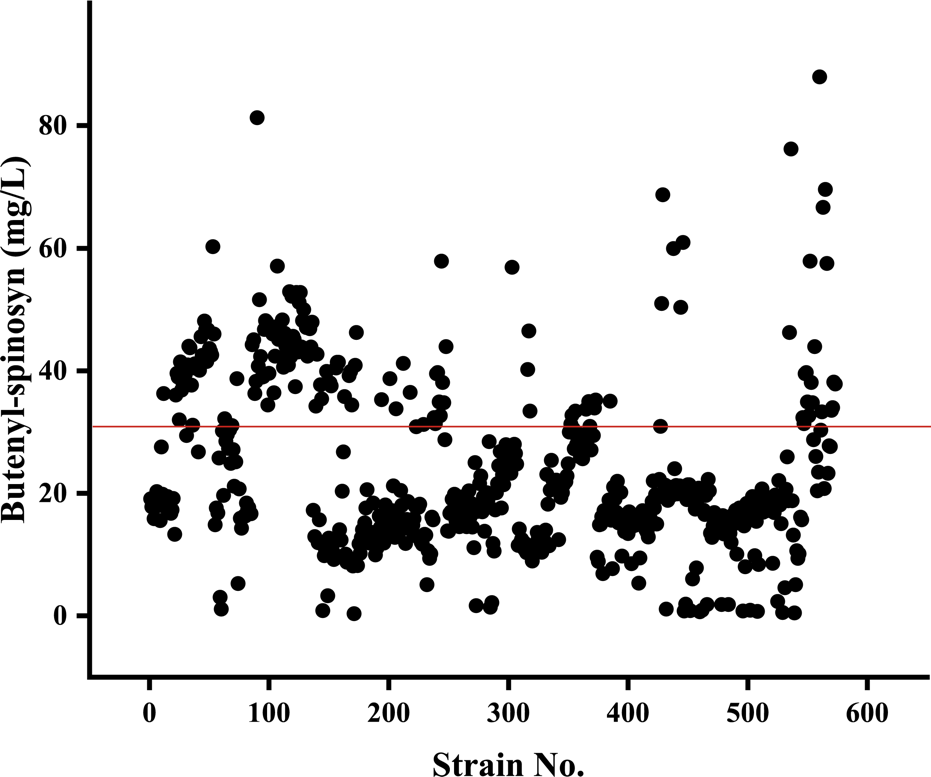


**Fig. S6** Effect of ARTP on strain mutation, and positive mutation rates.


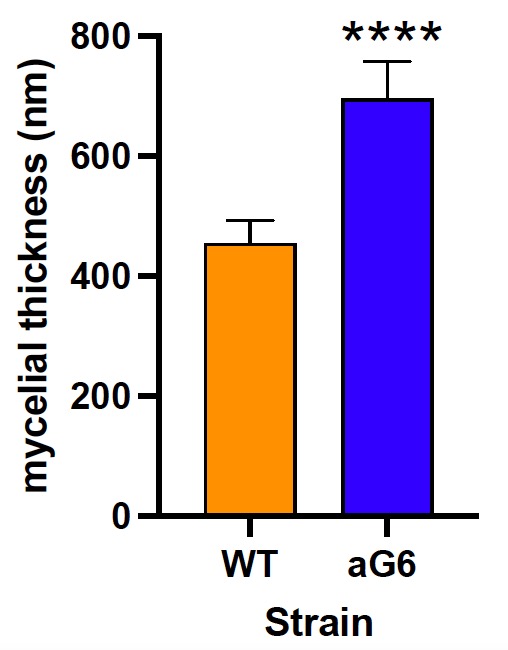


**Fig. S7** The mycelial thickness of the aG6 and WT. ****indicates P < 0.0001 compared to WT under the same conditions.


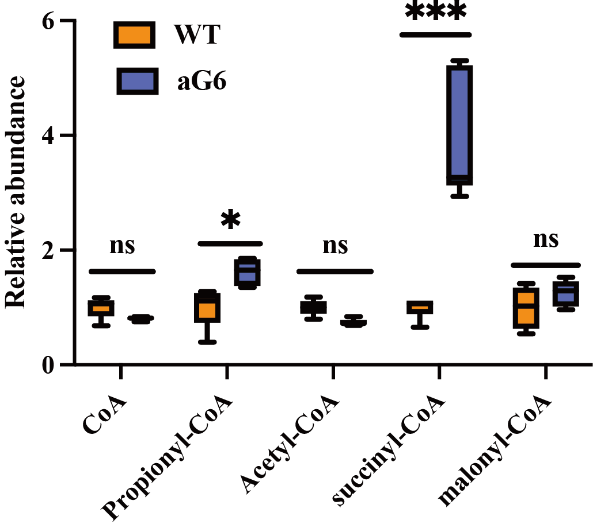


**Fig. S8.** Intracellular coenzyme A in WT and aG6 at 36h.

**Table S1.** Primers used in this study.

| Primer name | Sequence (5′-3′) |
| --- | --- |
| qbusA-F | gcttgcaaacgtattgggtatc |
| qbusA-R | actcggtattgcgtgttctc |
| qbusD-F | aaacgcccgaagcactat |
| qbusD-R | caagcgaatacccgacatct |
| qbusF-F | cgaggtcgtcaaacgagaag |
| qbusF-R | gtcctcacaagcgaggattt |
| qbusG-F | agctctgcacgacattagac |
| qbusG-R | agctgcgctttcccatt |
| qbusI-F | atcgacggcctgcattatc |
| qbusI-R | cgaataccaggttgtggtagaa |
| qbusJ-F | gtgtccgggtagttgatgtaag |
| qbusJ-R | cctggattcgcggtttctac |
| qbusO-F | atgatcgaaccgc |
| qbusO-R | tcgaattgtaaccagcctctac |
| qbusP-F | agttcgcacttcttcaacct |
| qbusP-R | ggtgaccatcgacaccatatt |
| FhrdB-rt | aggtgatcaacaagctc |
| RhrdB-rt | cggggtgatgtccatttc |
| qbldD-F | cagcagcagggtctgtc |
| qbldD-R | cgctccaggttgatcacaa |
| qssgA-F | agatctgcatgaagctcaacg |
| qssgA-R | caagaactccgcaagctgat |
| qwhiB2-F | acggacgaagaacaggaatg |
| qwhiB2-R | ttgagcttccggcgttc |

**Table S2.** The components of different media.

| **Media name** | **Composition (g/L)** |
| --- | --- |
| SP media | glucose10, soluble starch 10, yeast extract 5, N-Z-Amine 5, CaCO_3_ 1, agar 20, pH 7.5 |
| Seed media | Glucose 10，MgSO_4_·7H_2_O 2, KH_2_PO_4_ 0.5，Yeast extract 30，Peptonized milk 30, pH 7.5 |
| Fermentation media | gram per liter: glucose 26.4, cottonseed protein 20, NaCl 3, K_2_HPO_4_ 0.262, FeSO_4_ .7H_2_O 0.05, CaCO_3_ 1, pH 7.2 |

**Table S3.** General features of the *S. pogona* aG6 and WT genome.

| **Component of the genome** | **WT** | **aG6** |
| --- | --- | --- |
| Length | 8,199,208 bp | 7,768,050 bp |
| G + C content | 67.97% | 69.12% |
| Coding sequences | 8672 | 7,363 |
| tRNA | 52 | 52 |
| rRNA | 15 | 15 |
| Average CDS length | 817 | 837 |

**Table S4.** The number of genes affected by different SNPs and InDels in the mutant strain aG6.

| **Total SNPs** | 29 | **Total InDels** | 69 |
| --- | --- | --- | --- |
| In promoters | 0 | Insertion | 37 |
| In terminators | 0 | Deletion | 32 |
| In the CDS | 28 | In the CDS | 69 |
| Intergenic | 1 | Intergenic | 26 |
| Synonymous | 11 |  |  |
| Nonsynonymous | 17 |  |  |

**Table S5.** Mutated proteins in *S. pogona* aG6.

| **Gene NO. in *S. pogona* WT** | **Putative function** | **Variations** | **Effects on protein** | |
| --- | --- | --- | --- | --- |
| 1_orf05513 | IS1380 family transposase | SNP | | A256V |
| 1_orf06188 | IS110 family transposase | SNP | | I167V |
| 1_orf06840 | hypothetical protein | SNP | | G535V |
| 1_orf08793 | dTDP-glucose 4,6-dehydratase | SNP | | S233F |
| 1_orf01068 | NAD-glutamate dehydrogenase | SNP | | D1359N |
| 1_orf13359 | response regulator transcription factor | SNP | | V2A |
| 1_orf12469 | hypothetical protein | SNP | | G876D |
| 1_orf12154 | class I SAM-dependent methyltransferase | SNP | | R211P |
| 1_orf12152 | methyltransferase domain-containing protein | SNP | | G214R |
| dapE2 | SDR family NAD(P)-dependent oxidoreductase | SNP | | T1117I |
| 1_orf10840 | IS1182 family transposase | SNP | | W302C |
| 1_orf03210 | alcohol dehydrogenase catalytic domain-containing protein | SNP | | G291S |
| 1_orf05220 | helix-turn-helix transcriptional regulator | SNP | | G169E |
| sbnB | ornithine cyclodeaminase | Deletion | | Frameshift |
| 1_orf04012 | hypothetical protein SACE_6710 | Insertion | | Amino acid insertion |
| 1_orf04012 | hypothetical protein SACE_6710 | Deletion | | Frameshift |
| 1_orf03286 | protease inhibitor protein | Insertion | | Frameshift |
| 1_orf11537 | hypothetical protein | Deletion | | Amino acid insertion |
| 1_orf11537 | hypothetical protein | Deletion | | Frameshift |
| 1_orf11537 | hypothetical protein | Insertion | | Frameshift |
| 1_orf11537 | hypothetical protein | Insertion | | Amino acid insertion |
| 1_orf10715 | cysteine synthase A | Deletion | | Frameshift |
| griI | 2-amino-4, 5-dihydroxy-6-oxo-7-(phosphonooxy)heptanoate synthase | Insertion | | Amino acid insertion |
| 1_orf10769 | hypothetical protein | Insertion | | Amino acid insertion |
| griJ | putative aspartokinase homologue | Insertion | | Amino acid insertion |
| 1_orf11205 | FAD dependent oxidoreductase family protein | Insertion | | Frameshift |
| 1_orf03211 | subunit of transposase | Deletion | | Frameshift |
| 1_orf11149 | hypothetical protein | Insertion | | Frameshift |
| 1_orf11149 | hypothetical protein | Deletion | | Frameshift |
| 1_orf11591 | protein-L-isoaspartate(D-aspartate)O-methyltrans ferase | Insertion | | Frameshift |
| 1_orf10874 | geranylgeranyl diphosphate synthase, type I | Insertion | | Amino acid insertion |
| 1_orf00818 | GntR family transcriptional regulator, transcriptional repressor for pyruvate dehydrogenase complex | Deletion | | Frameshift |
| 1_orf12640 | NAD-dependent deacetylase | Deletion | | Frameshift |
| 1_orf01918 | XRE family transcriptional regulator | Insertion | | Amino acid insertion |
| 1_orf11087 | alanine-glyoxylate transaminase / serine-glyoxylate transaminase / serine-pyruvate transaminase | Deletion | | Frameshift |
| sbnD | siderophore biosynthesis protein SbnD | Deletion | | Frameshift |
| 1_orf00692 | putative UDP-glucose/GDP-mannose dehydrogenase | Deletion | | Frameshift |
| 1_orf11436 | CBS domain-containing protein | Deletion | | Frameshift |
| 1_orf11436 | CBS domain-containing protein | Insertion | | Frameshift |
| 1_orf10170 | formyl-CoA transferase | Deletion | | Frameshift |
| pstC2 | phosphate transport system permease protein | Deletion | | Deletion |
| pstC2 | phosphate transport system permease protein | Insertion | | Amino acid insertion |
| 1_orf05064 | RNA-directed DNA polymerase | Insertion | | Frameshift |
| nos | nitric-oxide synthase, bacterial | Insertion | | Frameshift |
| groES2 | chaperonin GroES | Insertion | | Frameshift |
| groES2 | chaperonin GroES | Deletion | | Frameshift |
| 2_orf00712 | hypothetical protein | Deletion | | Deletion |

**Table S6.** Mutant genes with SNPs and Indels in the CDS region of *S. pogona* aG6. Indel type: “I” for insertion, “D” for deletion.

| **Position** | **Ref_base<->sample _base/ InDel_seq (Indel type)** | **gene_startgene_end** | **Gene_id** |
| --- | --- | --- | --- |
| 5114144 | C<->A | 5114272-5115615 | sp.wt_GM004835 |
| 624947 | GTCC(I) | 623878-624822 | sp.wt_GM000569 |
| 3398707 | C(D) | 3398236-3398496 | sp.wt_GM003121 |
| 4360498 | C(D) | 4358973 -4359173 | sp.wt_GM004096 |
| 4362623 | GA(I) | 4365028-4366728 | sp.wt_GM004097 |
| 4363118 | A(D) | 4365028-4366728 | sp.wt_GM004097 |
| 4363190 | GGG (I) | 4365028-4366728 | 4365028 - 4366728 |
| 4401462 | C(I) | 4399771-4401426 | sp.wt_GM004131 |
| 4898665 | G(I) | 4893888-4897580 | sp.wt_GM004617 |
| 4901647 | TG(I) | 4903365-4904117 | sp.wt_GM004618 |
| 4902649 | G(I) | 4903365-4904117 | sp.wt_GM004618 |
| 5896802 | C(D) | 5896811-5897122 | sp.wt_GM005547 |
| 5957036 | C(D) | 5957264-5957605 | sp.wt_GM005594 |
| 6303654 | T (I) | 6301368-6303650 | sp.wt_GM005870 |
| 6354085 | GC(D) | 6354183-6355535 | sp.wt_GM005915 |
| 6770765 | CG(I) | 6770473-6770661 | sp.wt_GM006326 |
| 6776766 | GCGG(I) | 6776818-6778110 | sp.wt_GM006334 |
| 7057711 | GG(I) | 7057497-7057694 | sp.wt_GM006631 |
| 37494 | TGA(I) | 37838-50533 | sp.wt_GM007367 |
| 314440 | AG(D) | 311035-312309 | sp.wt_GM007625 |
| 315939 | G(D) | 318197-319450 | sp.wt_GM007626 |
| 337705 | ATCG(I) | 336864-33763 | sp.wt_GM007644 |
| 355678 | C(D) | 354827 - 355525 | sp.wt_GM007658 |
| 406517 | C(I) | 406645-406815 | sp.wt_GM007716 |
| 406879 | G(I) | 406645-406815 | sp.wt_GM007716 |
| 419788 | G(D) | 417817-419670 | sp.wt_GM007731 |
| 423772 | G(D) | 423845-423982 | sp.wt_GM007736 |

**Table S7.** Comparative transcriptional profile analysis of genes at day 6.

| **Gene_id** | **KO name** | **Gene description** | **Log2FC(WT_6d/aG6_6d)** | **P value** |
| --- | --- | --- | --- | --- |
| **Comparative transcriptional profile analysis of genes in ribosomal proteins** | | | | |
| 1_orf04261 | rplJ | large subunit ribosomal protein L10 | -3.4171939 | 1.49E-29 |
| 1_orf04259 | rplL | large subunit ribosomal protein L7/L12 | -3.6613441 | 3.11E-26 |
| 1_orf04181 | rplE | large subunit ribosomal protein L5 | -2.5464413 | 9.89E-24 |
| 1_orf04183 | rplN | large subunit ribosomal protein L14 | -2.7625979 | 9.08E-22 |
| 1_orf04168 | rplO | large subunit ribosomal protein L15 | -2.569238 | 6.66E-21 |
| 1_orf04187 | rplP | large subunit ribosomal protein L16 | -2.3847821 | 1.92E-20 |
| 1_orf04189 | rpsC | small subunit ribosomal protein S3 | -2.4818875 | 5.33E-20 |
| 1_orf04177 | rpsH | small subunit ribosomal protein S8 | -2.2703429 | 3.27E-19 |
| 1_orf04191 | rpsS | small subunit ribosomal protein S19 | -2.2612442 | 4.66E-19 |
| 1_orf04184 | rplX | large subunit ribosomal protein L24 | -2.5387748 | 1.36E-18 |
| 1_orf04185 | rpsQ | small subunit ribosomal protein S17 | -1.7261954 | 5.59E-18 |
| 1_orf04176 | rplF | large subunit ribosomal protein L6 | -2.3581128 | 1.03E-17 |
| 1_orf04190 | rplV | large subunit ribosomal protein L22 | -2.4787733 | 4.22E-17 |
| 1_orf04172 | rpsE | small subunit ribosomal protein S5 | -2.5779558 | 5.13E-17 |
| 1_orf04195 | rplW | large subunit ribosomal protein L23 | -2.6146665 | 6.78E-16 |
| 1_orf04960 | rplI | large subunit ribosomal protein L9 | -2.062654 | 1.16E-15 |
| 1_orf04148 | rplQ | large subunit ribosomal protein L17 | -2.1910342 | 2.64E-15 |
| 1_orf04194 | rplB | large subunit ribosomal protein L2 | -2.3130216 | 1.32E-14 |
| 1_orf04179 | rpsN | small subunit ribosomal protein S14 | -2.7860686 | 2.46E-13 |
| 1_orf04174 | rplR | large subunit ribosomal protein L18 | -2.094681 | 3.25E-13 |
| 2_orf00490 | rplT | large subunit ribosomal protein L20 | -1.9260178 | 7.99E-13 |
| 1_orf04198 | rplD | large subunit ribosomal protein L4 | -2.4330787 | 8.05E-13 |
| 1_orf04200 | rplC | large subunit ribosomal protein L3 | -2.6007677 | 1.23E-11 |
| 1_orf04150 | rpsD | small subunit ribosomal protein S4 | -2.2108849 | 2.44E-11 |
| 1_orf02879 | rpmB | large subunit ribosomal protein L28 | -2.0944744 | 7.40E-11 |
| 1_orf04962 | rpsR | small subunit ribosomal protein S18 | -2.1400027 | 8.75E-11 |
| 1_orf04186 | rpmC | large subunit ribosomal protein L29 | -1.5948886 | 1.20E-10 |
| 1_orf04155 | rpmJ | large subunit ribosomal protein L36 | -2.366657 | 1.23E-10 |
| 1_orf03154 | rpmE | large subunit ribosomal protein L31 | -2.1195128 | 2.76E-10 |
| 1_orf04153 | rpsM | small subunit ribosomal protein S13 | -2.0969586 | 3.76E-10 |
| 2_orf00489 | rpmI | large subunit ribosomal protein L35 | -1.7033372 | 9.63E-10 |
| 1_orf04114 | rpsI | small subunit ribosomal protein S9 | -2.2553302 | 3.12E-09 |
| 1_orf04965 | rpsF | small subunit ribosomal protein S6 | -1.7461486 | 5.44E-09 |
| 1_orf04264 | rplA | large subunit ribosomal protein L1 | -1.309284 | 9.72E-09 |
| 1_orf02737 | rpsP | small subunit ribosomal protein S16 | -2.0333039 | 1.56E-08 |
| 1_orf07925 | rpmA | large subunit ribosomal protein L27 | -2.1626147 | 1.89E-08 |
| 1_orf04266 | rplK | large subunit ribosomal protein L11 | -1.2058126 | 1.40E-07 |
| 1_orf02730 | rplS | large subunit ribosomal protein L19 | -1.750932 | 2.42E-07 |
| 1_orf09251 | rpsA | small subunit ribosomal protein S1 | -1.2636422 | 2.55E-07 |
| 1_orf07970 | rpsT | small subunit ribosomal protein S20 | -1.4704576 | 2.67E-07 |
| 1_orf04202 | rpsJ | small subunit ribosomal protein S10 | -2.0318654 | 2.92E-07 |
| 1_orf04152 | rpsK | small subunit ribosomal protein S11 | -1.818812 | 3.54E-07 |
| 1_orf04115 | rplM | large subunit ribosomal protein L13 | -1.9699396 | 6.71E-07 |
| 1_orf04208 | rpsL | small subunit ribosomal protein S12 | -1.811284 | 8.70E-07 |
| 1_orf05291 | rpmH | large subunit ribosomal protein L34 | -2.4295444 | 9.84E-07 |
| 1_orf06716 | rplY | large subunit ribosomal protein L25 | -1.4792718 | 6.91E-06 |
| 1_orf04206 | rpsG | small subunit ribosomal protein S7 | -1.5912257 | 7.68E-06 |
| 1_orf04275 | rpmG | large subunit ribosomal protein L33 | -1.2007009 | 9.09E-06 |
| 1_orf02690 | rpsB | small subunit ribosomal protein S2 | -1.3271218 | 9.24E-06 |
| 1_orf07923 | rplU | large subunit ribosomal protein L21 | -1.7104501 | 2.54E-05 |
| 1_orf04171 | rpmD | large subunit ribosomal protein L30 | -1.7971936 | 2.84E-05 |
| 1_orf02819 | rpmF | large subunit ribosomal protein L32 | -1.4236007 | 2.90E-05 |
| **Comparative transcriptional profile analysis of genes in valine, leucine and isoleucine degradation** | | | | |
| 1_orf05171 | bkdB | dihydrolipoamide acetyltransferase | -1.9603767 | 3.06E-08 |
| 1_orf04535 | acdH | acyl-CoA dehydrogenase | -1.3167296 | 4.48E-08 |
| 1_orf05176 | bkdA1 | pyruvate dehydrogenase E1 component alpha subunit | -1.2786164 | 3.89E-07 |
| 1_orf05175 | bkdA2 | pyruvate dehydrogenase E1 component beta subunit | -1.1782113 | 1.95E-05 |
| 1_orf00879 | bkdA | 2-oxoisovalerate dehydrogenase E1 component | -1.110256 | 0.00013326 |
| **Comparative transcriptional profile analysis of genes in RNA polymerase** | | | | |
| 1_orf04248 | rpoC | DNA-directed RNA polymerase subunit beta | -1.55662 | 2.65E-11 |
| 1_orf04149 | rpoA | DNA-directed RNA polymerase subunit alpha | -1.79775 | 7.72E-10 |
| 1_orf04250 | rpoB | DNA-directed RNA polymerase subunit beta | -1.20565 | 1.51E-07 |
| 2_orf00064 | rpoZ | DNA-directed RNA polymerase subunit omega | -0.86192 | 0.00928 |
| **Comparative transcriptional profile analysis of genes in TCA cycle** | | | | |
| 1_orf00954 | korA | 2-oxoglutarate/2-oxoacid ferredoxin oxidoreductase subunit alpha | -1.54846 | 7.59E-08 |
| 1_orf00953 | korB | 2-oxoglutarate/2-oxoacid ferredoxin oxidoreductase subunit beta | -1.33099 | 9.86E-08 |
| 1_orf12664 | acnA | aconitate hydratase | -1.33684 | 1.09E-07 |
| 1_orf03853 | icd | isocitrate dehydrogenase | -1.00237 | 2.91E-05 |
| 1_orf07040 | fumC | fumarate hydratase%2C class II | 1.277273 | 0.004349 |
| 1_orf12478 | mdh | malate dehydrogenase | 0.86267 | 0.006288 |
| 1_orf03777 | sdhA | succinate dehydrogenase / fumarate reductase%2C flavoprotein subunit | -0.55778 | 0.064311 |
| 1_orf06427 | gltA | citrate synthase | 0.34589 | 0.185609 |
| 1_orf03907 | sucD | succinyl-CoA synthetase alpha subunit | -0.46929 | 0.249767 |
| 1_orf03780 | sdhB | succinate dehydrogenase / fumarate reductase%2C iron-sulfur subunit | -0.37452 | 0.26226 |
| 1_orf06445 | gltA | citrate synthase | -0.34526 | 0.324957 |
| 1_orf07429 | sdhA | succinate dehydrogenase / fumarate reductase%2C flavoprotein subunit | -0.29875 | 0.331714 |
| 1_orf03352 | kgd | 2-oxoglutarate decarboxylase | -0.1986 | 0.36921 |
| 1_orf03775 | sdhD | succinate dehydrogenase / fumarate reductase%2C membrane anchor subunit | 0.247737 | 0.469238 |
| 1_orf07431 | sdhB | succinate dehydrogenase / fumarate reductase%2C iron-sulfur subunit | -0.20296 | 0.517152 |
| 1_orf03910 | sucC | succinyl-CoA synthetase beta subunit | -0.21547 | 0.577838 |
| 1_orf03773 | sdhC | succinate dehydrogenase / fumarate reductase%2C cytochrome b subunit | 0.19157 | 0.621211 |
| 1_orf08796 | fumA | fumarate hydratase%2C class I | -0.0944 | 0.772864 |
| **Comparative transcriptional profile analysis of genes in oxidative phosphorylation** | | | | |
| 1_orf07437 | coxA | cytochrome c oxidase subunit I | -1.2861209 | 9.97E-10 |
| 1_orf04314 | nuoJ | NADH-quinone oxidoreductase subunit J | -1.5194408 | 2.39E-09 |
| 1_orf03133 | atpA | F-type H%2B-transporting ATPase subunit alpha | -1.5288357 | 1.85E-08 |
| 1_orf04320 | nuoG | NADH-quinone oxidoreductase subunit G | -1.5517882 | 4.51E-08 |
| 1_orf03137 | 1_orf03137 | F-type H%2B-transporting ATPase subunit b | -1.6974639 | 2.72E-07 |
| 1_orf08613 | coxB | cytochrome c oxidase subunit II | -1.9639985 | 4.13E-07 |
| 1_orf08627 | qcrB | ubiquinol-cytochrome c reductase cytochrome b subunit | -1.3268729 | 1.50E-06 |
| 1_orf04304 | nuoN | NADH-quinone oxidoreductase subunit N | -1.3570018 | 3.84E-06 |
| 1_orf03129 | atpD | ATPase subunit beta | -1.108371 | 7.92E-06 |
| 1_orf04324 | nuoD | NADH-quinone oxidoreductase subunit D | -1.1024183 | 8.04E-06 |
| 1_orf03141 | atpB | F-type H%2B-transporting ATPase subunit a | -1.4631845 | 9.74E-06 |
| 1_orf03138 | atpE | ATPase subunit c | -1.4274457 | 1.32E-05 |
| 1_orf04325 | nuoE | NADH-quinone oxidoreductase subunit E | -1.156951 | 1.76E-05 |
| 1_orf03135 | atpH | F-type H%2B-transporting ATPase subunit delta | -1.3832431 | 2.06E-05 |
| 1_orf04329 | nuoB | NADH-quinone oxidoreductase subunit B | -1.0333366 | 2.72E-05 |
| 1_orf04327 | nuoC | NADH-quinone oxidoreductase subunit C | -1.1190749 | 3.75E-05 |
| 1_orf04323 | nuoF | NADH-quinone oxidoreductase subunit F | -1.2693043 | 3.75E-05 |
| 1_orf07344 | ndh | NADH dehydrogenase | 1.10102117 | 3.85E-05 |
| 1_orf04310 | nuoL | NADH-quinone oxidoreductase subunit L | -1.3239949 | 8.50E-05 |
| 1_orf04306 | nuoM | NADH-quinone oxidoreductase subunit M | -1.1373767 | 0.00014943 |
| 1_orf04319 | nuoH | NADH-quinone oxidoreductase subunit H | -1.2205958 | 0.00031536 |
| 1_orf04317 | nuoI | NADH-quinone oxidoreductase subunit I | -1.0724959 | 0.00076797 |
| 1_orf04316 | nuoK | NADH-quinone oxidoreductase subunit K | -1.0785546 | 0.00173165 |
| 1_orf08623 | qcrC | ubiquinol-cytochrome c reductase cytochrome c subunit | -1.0020957 | 0.00173828 |
| 1_orf00273 | ntpK | ATPase subunit K | 1.35963567 | 0.01352781 |

**Table S8.** Comparative transcriptional profile analysis of genes at day 3.

| **Gene_id** | **KO name** | **Gene description** | **Log2FC(WT-3d/aG6-3d)** | **P value** |
| --- | --- | --- | --- | --- |
| 1_orf00953 | korB | 2-oxoacid ferredoxin oxidoreductase subunit beta | -0.6871942 | 3.50E-05 |
| 1_orf00954 | korA | 2-oxoacid ferredoxin oxidoreductase subunit alpha | -0.9438166 | 5.98E-10 |
| \| \| 1_orf08042 \| \| --- \| \| \| --- \| --- \| | mmsA | pyruvate dehydrogenase E1 component alpha subunit | -0.5170521 | 0.00543781 |
| \| \| \| 1_orf04537 \| \| --- \| \| \| --- \| --- \| \| \| --- \| --- \| --- \| | bccA | pyruvate dehydrogenase E1 component beta subunit | -1.9161913 | 5.05E-21 |
| 1_orf02121 | lpd | dihydrolipoamide dehydrogenase | -1.958958 | 3.02E-16 |
| 1_orf02879 | rpmB | large subunit ribosomal protein L28 | -0.7003489 | 0.00076212 |
| 2_orf00490 | rplT | large subunit ribosomal protein L20 | -0.3453017 | 0.0785878 |
| 1_orf05291 | rpmH | large subunit ribosomal protein L34 | -0.4660878 | 0.07904011 |
| 1_orf02819 | rpmF | large subunit ribosomal protein L32 | -0.2339748 | 0.25954803 |
| 1_orf04275 | rpmG | large subunit ribosomal protein L33 | -0.1830221 | 0.28728002 |
| 1_orf03154 | rpmE | large subunit ribosomal protein L31 | -0.1797309 | 0.30994406 |
| 2_orf00489 | rpmI | large subunit ribosomal protein L35 | -0.1613651 | 0.43979888 |
| 1_orf02730 | rplS | large subunit ribosomal protein L19 | -0.0898085 | 0.66140323 |
| 1_orf04115 | rplM | large subunit ribosomal protein L13 | -0.0511365 | 0.80477449 |
| 1_orf07925 | rpmA | large subunit ribosomal protein L27 | -0.0381265 | 0.86537856 |
| 1_orf04535 | acdH | acyl-CoA dehydrogenase | -2.0597979 | 1.85E-22 |

**Nomenclature**

| **Abbreviation** | **Description** |
| --- | --- |
| 2-Hydroxyphenylacetate | 2-HP |
| 2-Oxepin-2(3H)-ylideneacetul-CoA | 2-OP |
| 3-Oxo-5,6-didehydrosuberyl-CoA | 3-OA |
| 4-Methyl-2-oxopentanoate | 4-MO |
| 3-Methyl-butanoyl-dihydrolipoamide-E | 3-ME |
| 3-Methylbutanoyl-CoA | 3-MA |
| 3-Methylbut-2-enoyl-CoA | 3-MeA |
| 3-Methyl-2-oxobutanoate | 3-MO |
| 2-Methyl-butanoyl-dihydrolipoamide-E | 2-ME |
| 3-Hydroxy-isobutyry-CoA | 3-HA |
| Methylmalonate semialdehyde | MS |
| 3-Methyl-2-oxopentanoate | 3-M-2-OP |
| 2-Methyl-butanoyl-CoA | 2-MBC |
| tran-2-Methyl-but-2-enoyl-CoA | T-2-MA |
| 3-Hydroxy-2-methylbutyryl-CoA | 3-H-2-MA |
| 2-Methyl-acetoacetyl-CoA | 2-MAA |
